# Optimizing oncology drug development: systematic review of 22 years of myeloma randomized controlled trials

**DOI:** 10.1093/jnci/djaf326

**Published:** 2025-11-09

**Authors:** Maria Mainou, Muatassem Alsadhan, Kalliopi Tsapa, Alissa Visram, Hira Mian, Rakesh Popat, Elias K Mai, Rajshekhar Chakraborty, Samer Al Hadidi, Meera Mohan, Aniko Szabo, Oliver Van Oekelen, Edward R Scheffer Cliff, Ghulam Rehman Mohyuddin

**Affiliations:** Clinical Research and Evidence-Based Medicine Unit, Second Medical Department, Aristotle University of Thessaloniki, Thessaloniki, Greece; Division of Hematology, Huntsman Cancer Institute, University of Utah, Salt Lake City, UT, United States; Clinical Research and Evidence-Based Medicine Unit, Second Medical Department, Aristotle University of Thessaloniki, Thessaloniki, Greece; Division of Hematology, McMaster University, Hamilton, ON, Canada; Division of Hematology, McMaster University, Hamilton, ON, Canada; Division of Hematology, University College London, London, United Kingdom; Heidelberg Myeloma Center, Internal Medicine V, Hematology, Oncology and Rheumatology, Heidelberg University Hospital and Medical Faculty Heidelberg, Heidelberg, Germany; Division of Hematology/Oncology, Columbia University Medical Center, New York City, NY, United States; Department of Internal Medicine, UT Southwestern Medical Center, Dallas, TX, United States; Division of Hematology/Oncology, Department of Medicine, Medical College of Wisconsin, Milwaukee, WI, United States; Division of Hematology/Oncology, Department of Medicine, Medical College of Wisconsin, Milwaukee, WI, United States; Department of Medicine, Icahn School of Medicine at Mt Sinai, New York City, NY, United States; Program On Regulation, Therapeutics and Law, Division of Pharmacoepidemiology and Pharmacoeconomics, Department of Medicine, Brigham and Women’s Hospital, Boston, MA, United States; Department of Clinical Haematology, Peter MacCallum Cancer Centre and Royal Melbourne Hospital, Melbourne, Australia; Sir Peter MacCallum Department of Oncology, The University of Melbourne, Melbourne, Australia; Division of Hematology, Huntsman Cancer Institute, University of Utah, Salt Lake City, UT, United States

## Abstract

**Background:**

Although myeloma represents a key success story in oncology, some drugs have failed to meet primary endpoints in randomized controlled trials (RCTs), despite promising early phase activity. This analysis aimed to understand factors that increase the likelihood of meeting primary endpoints in myeloma RCTs.

**Methods:**

Myeloma RCTs published through October 2023 were identified using MEDLINE, PubMed, Embase, and the Cochrane Registry. Studies were classified as head-to-head (substituting 1 regimen for another) or add-on (adding 1 drug to existing regimen). Trials were considered successful if they achieved statistical significance for primary outcomes. Logistic regression identified predictors of meeting trial endpoints.

**Results:**

A total of 145 comparisons from 123 RCTs were included. Only 2 factors were independently associated with meeting primary endpoints in multivariate analysis. Higher median participant age was associated with lower odds of meeting the primary endpoint (odds ratio [OR] per 1-year increase = 0.90, 95% confidence interval [CI] = 0.83 to 0.98). Overall survival (OS) was the primary endpoint in 20 of 145 comparisons, of which 3 of 20 met their endpoint. Selecting OS as primary endpoint was associated with reduced likelihood of success compared with progression-free survival by 94% (OR = 0.06, 95% CI = 0.01 to 0.23). Head-to-head design was not associated with lower success rates than add-on design (OR = 0.59; 95% CI = 0.22 to 1.62).

**Conclusion:**

Two key factors predicted higher likelihood of meeting endpoints: younger patient age and primary endpoints other than OS. Although head-to-head design is considered riskier, it was not associated with decreased success. This analysis aims to better inform clinicians, industry, and regulators in myeloma drug development.

## Introduction

Multiple myeloma represents a remarkable success in oncology. With more than 19 novel drug approvals in the past 2 decades, the use of highly effective treatment combinations has changed the natural history of the disease and extended the median overall survival (OS) from approximately 2-3 years to more than 10 years.^1^^,^^2^ Prior to the 21st century and the development of these novel therapies, steroids and alkylators were the mainstay of myeloma treatment.^1^ Progress accelerated with the discovery of immunomodulatory drugs (IMiDs) thalidomide, and subsequently lenalidomide and pomalidomide,^3^ followed by the development of proteasome inhibitors bortezomib and carfilzomib, and more recently, anti-CD38 antibodies daratumumab and isatuximab.^4^ The discovery of further immunotherapies, such as bispecific antibodies and chimeric antigen receptor T-cells, as well as additional targets, such as B-cell maturation antigen^5^ and G protein–coupled receptor class C group 5 member D,^6^ offer further opportunities to prolong the duration of remission—and hopefully lives—for patients living with myeloma.

Such progress in myeloma is the result of both highly effective basic scientific discovery and clinical development programs in myeloma, and the collective effort of scientists, industry, clinical researchers, and of course patients. The use of various regulatory pathways, flexibilities, and incentives^7^ designed to speed up drug development has also helped lubricate the process of drug development in oncology and myeloma. Although many drugs have shown promising activity—the ability to reduce myeloma burden as measured by serum M-protein or free light chains—in early phase, typically single-arm studies, the ultimate determinant of a drug’s success is its ability to demonstrate improved clinical outcomes for patients in randomized controlled trials (RCTs).^8^ However, some recent treatments have failed to meet their primary endpoint in RCTs, despite demonstrating promising activity and responses in single-arm studies,^9^^,^^10^ leading to withdrawal of the drugs,^11^ concerns regarding the potential for unproven drugs to cause harm, and the optimal approach to trial design.^12-14^

An important aspect of this concern is the shift from clinical endpoints, such as OS, toward surrogate measures. This evolution raises broader questions about how endpoint selection and trial design influence the likelihood that pivotal myeloma trials meet their prespecified goals, and offer patients a net clinical benefit. In the light of the rapid expansion of phase 3 myeloma trials, both successful and unsuccessful, a systematic evaluation of trial and design-level factors associated with success is needed. A better understanding of these predictive factors can assist in the design and interpretation of future studies. In this study, we aimed to better characterize drug development in oncology by analyzing myeloma RCTs over the past 22 years and identifying drug- and trial design characteristics linked to studies achieving their primary endpoints, with a particular focus on whether add-on trials are more likely to be associated with positive results than substitution trials.

## Methods

### Search strategy

This study was based on a previously published systematic review,^15^ the protocol for which is registered in the Open Science Framework. This report follows the PRISMA guidelines for reporting.^16^ As the data were publicly available and did not involve human participant research, Institutional Review Board review was not required. We identified RCTs in myeloma by searching MEDLINE/PubMed, Embase, and the Cochrane Registry of RCTs. The search strategy (Supplementary Methods) incorporated free-text terms related to myeloma, along with relevant MeSH terms, and filtered specifically for RCTs published in English.

### Study inclusion criteria

We included randomized phase 3 studies with a published primary analysis from January 1, 2001 (when IMiDs and proteasome inhibitors emerged), to October 17, 2023. Two independent reviewers screened titles, abstracts, and full texts to identify eligible trials, and extracted data using standardized online forms (DistillerSR). Discrepancies were resolved by consensus, with a third reviewer consulted when necessary. As this study aimed to evaluate the predictors of success of different approaches to trial design in drug development, we excluded studies that compared conditioning regimens in transplant or transplant vs nontransplant approaches. Studies comparing induction regimens prior to transplantation and consolidation/maintenance strategies posttransplantation were included. We also excluded studies on smoldering myeloma, supportive care, and those comparing only the methods of administration or dosages. We conducted a subgroup analysis of trials leading to FDA approval using publicly available FDA data.^17^

### Study metrics and definitions

A trial was considered to have met its primary endpoint if it achieved a statistically significant result in its primary endpoint. Trials with co-primary endpoints were defined as meeting either of the endpoints described and categorized separately. For example, a trial with co-primary endpoints of progression-free survival (PFS) and OS would be categorized into 2 separate studies for analysis, with the likelihood of meeting the endpoint and variables influencing this likelihood analyzed for each endpoint. In cases in which a trial included multiple randomizations, each randomization was treated as an independent comparison and data were extracted separately.

Add-on trials were defined as those adding a drug to an existing treatment backbone (eg, comparing a 3-drug to a 2-drug combination), whereas substitution trials were defined as those in which 1 drug was replaced with another, keeping the number of agents in the control and intervention arms the same (eg, 3-drug vs 3-drug combinations). Studies comparing regimens with fewer drugs in the intervention arm (eg, a 3-drug control regimen vs a 2-drug novel regimen, such as melphalan/prednisone/thalidomide vs lenalidomide/dexamethasone, or ciltacabtagene autoleucel vs standard-of-care triplet therapy) were also classified as substitution trials. Studies with more than 2 arms were assessed based on a primary comparison.

We grouped together time-to-event progression outcomes (event-free survival, PFS, time-to-progression, or time-to-treatment failure), and response-based outcomes (response rate, measurable residual disease [MRD] negativity) for the purpose of this analysis. For descriptive analyses, trials were also grouped by study design (blinded or open label), funding source (pharmaceutical industry funded or nonindustry funded); year of publication (before/after 2010, before/after 2015), setting (newly diagnosed myeloma, maintenance or relapsed/refractory myeloma), study location (Europe, United States, rest of the world, or multicontinental), and transplant eligibility (eligible, ineligible, both, or unspecified).

No formal risk-of-bias assessment was conducted, as our objective was to identify associations between trial-level factors and trial success.

### Statistical analysis

The primary analysis was a multivariable logistic regression examining associations between trial-level factors and the likelihood of meeting the primary endpoint. A key secondary question included how substitution vs add-on trial designs affected this likelihood.

Univariate analyses were conducted at first, and inference was anchored in the prespecified multivariable model. Each study was treated as a primary sampling unit in complex-survey regression to account for potential nonindependence from multiple comparisons within a trial. Study design-related variables were selected for the inclusion in the multivariable model based primarily on substantive interest rather than univariable prescreening. Subgroup analyses by disease setting and by outcome type were also performed to ensure robustness of findings.

*P* < .05 was deemed statistically significant, and all *P*-values reported are 2-tailed. Regression results are presented as odds ratios (ORs) with 95% confidence intervals (CIs) and corresponding *P*-values. Continuous data are expressed as mean ± SD or as median with interquartile range (*Q*_1_–*Q*_3_), and categorical data are shown as frequencies (*n*) and percentages (%).

## Results

### Characteristics of included studies

**Figure 1.**
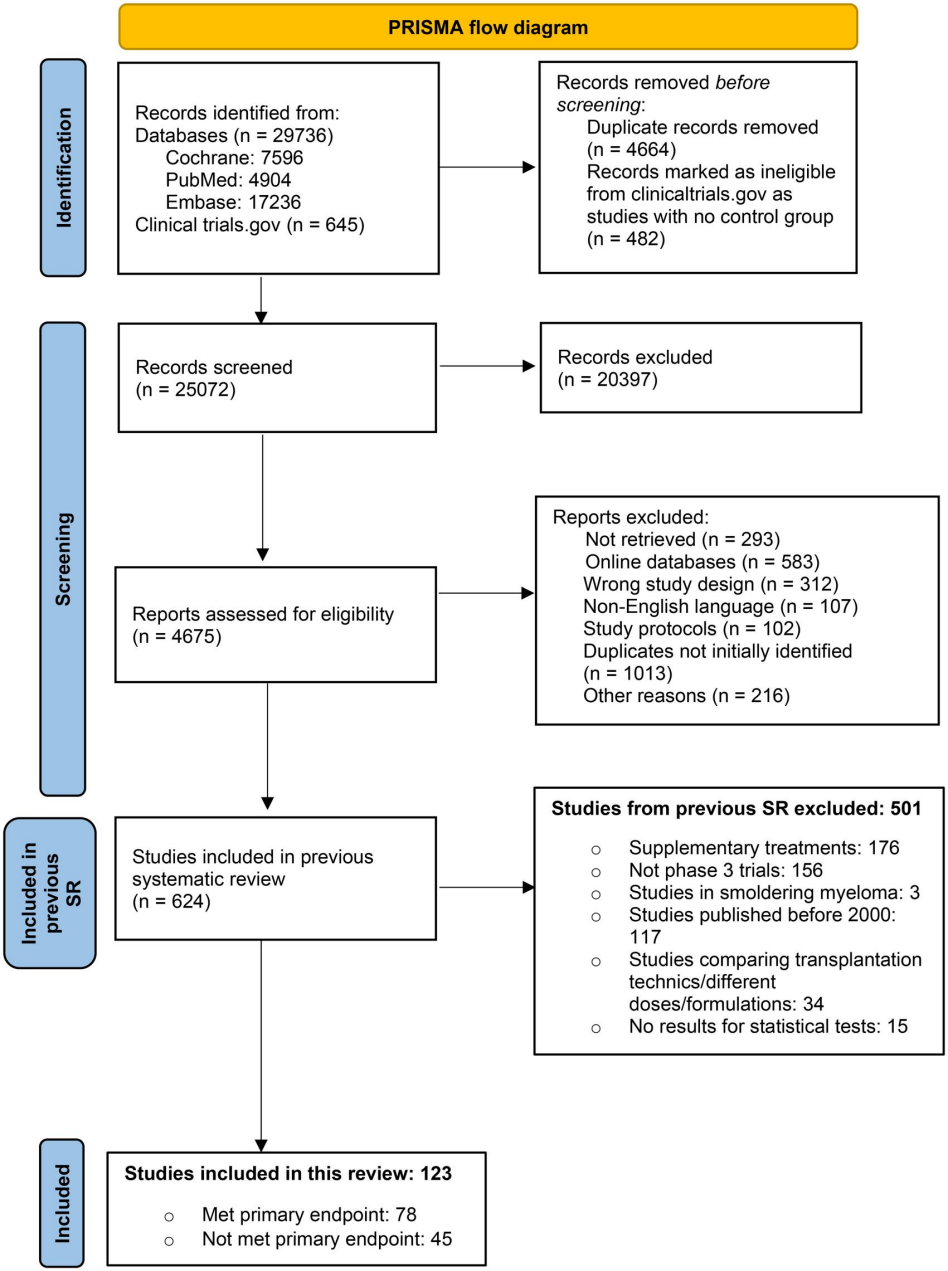
PRISMA flowchart depicting systematic study selection process.

We identified 123 phase 3 myeloma RCTs involving 145 treatment comparisons and 56 024 patients, accounting for multiple arms and randomizations (Figure 1). For consistency, all subsequent information was based on these comparisons. Among these, 84 of 145 (58%) met the primary endpoint. The characteristics of the included comparisons are highlighted in Table 1, and all comparisons are listed in Tables S1 and S2.

**Table 1.** Characteristics of the included studies.

Study characteristics	All comparisons, *N* = 145 (%)^a^	Comparisons that met endpoint, *n* = 84, *n* (%)	Comparisons that did not meet endpoint, *n* = 61, *n* (%)
Sample size—median (IQR)	353 (229-646)	460 (251-660)	306 (203-564)
Age—min—median (IQR)	38 (32-56)	35 (30-44)	50 (35-59)
Age—max—median (IQR)	84 (73-89)	83 (73-89)	84 (73-89)
Age—center—median (IQR)	65.0 (59.0-70.9)	63.5 (59.0-67.0)	66.0 (64.0-73.0)
Newly diagnosed setting, *N* (%)	103 (71)	55 (65)	48 (79)
Relapsed/refractory setting, *N* (%)	42 (29)	29 (35)	13 (21)
PFS as primary endpoint, *N* (%)	77 (53)	48 (57)	29 (48)
OS as primary endpoint, *N* (%)	20 (14)	3 (4)	17 (28)
Response as primary endpoint, *N* (%)	31 (21)	18 (21)	13 (21)
Proteasome inhibitor included, *N* (%)	61 (42)	40 (48)	21 (34)
IMID included, *N* (%)	99 (68)	59 (70)	40 (66)
Monoclonal antibody anti-CD38 included, *N* (%)	15 (10)	15 (18)	0 (0)
Industry funding, *N* (%)	108 (74)	70 (83)	38 (62)
Add-on trial, *N* (%)	104 (72)	64 (76)	40 (66)
Head-to-head trial, *N* (%)	41 (28)	20 (24)	21 (34)
North America based, *N* (%)	21 (14)	6 (7)	15 (25)
Europe based, *N* (%)	61 (42)	33 (39)	28 (46)
Rest of the world (%)	11 (8)	8 (10)	3 (5)
Multicontinental, *N* (%)	52 (36)	37 (44)	15 (25)
Published after 2010, *N* (%)	102 (70)	59 (70)	43 (70)
Double blind or assessors, *N* (%)	21 (14)	18 (21)	3 (5)
Open label or no report on blinding, *N* (%)	124 (86)	66 (79)	58 (95)

Abbreviations: IMiD = immunomodulatory drug; IQR = interquartile range; OS = overall survival; PFS = progression-free survival.

a One hundred twenty-three randomized controlled trials that included 145 treatment comparisons accounting for multiple arms and multiple randomization.

The median number of total patients randomized in all comparisons was 353 (229-646). Comparisons meeting their primary endpoint included a median of 460 patients (251-660), whereas comparisons that did not meet their primary endpoint included a median of 306 (203-564). A total of 103 of 145 comparisons (71%) were conducted in patients with newly diagnosed myeloma (28 of these 103 studied maintenance treatment postautograft), whereas 42 of 145 comparisons (29%) were in relapsed/refractory disease. Among all comparisons, 25 of 145 (17%) demonstrated OS improvement.

### Endpoint evaluation

Among these 145 comparisons, 107 (74%) were open label, 17 (12%) did not report blinding, 18 (12%) were double blind, and 3 (2%) reported only blinding of the outcome assessors. The majority (104, 72%) were add-on comparisons and the remaining 28% were substitution comparisons. The most common primary outcome was PFS in 77 of 145 (53%) comparisons, followed by response rate in 31 of 145 (21%) and OS in 20 of 145 (14%). Only 3 of these 20 OS primary comparisons (15%) met their endpoint. Overall survival was the sole primary outcome of the IFM01/01 trial comparing melphalan, prednisone, and thalidomide with melphalan and prednisone in newly diagnosed myeloma.^18^ In the other 2 studies, OS was the co-primary endpoint alongside PFS: 1 compared induction regimens^19^ and the other assessed thalidomide consolidation postautologous transplant.^20^ All 3 studies initiated enrollment during 2002-2003, concluded between 2005 and 2007, and were published prior to 2013.

**Figure 2.**
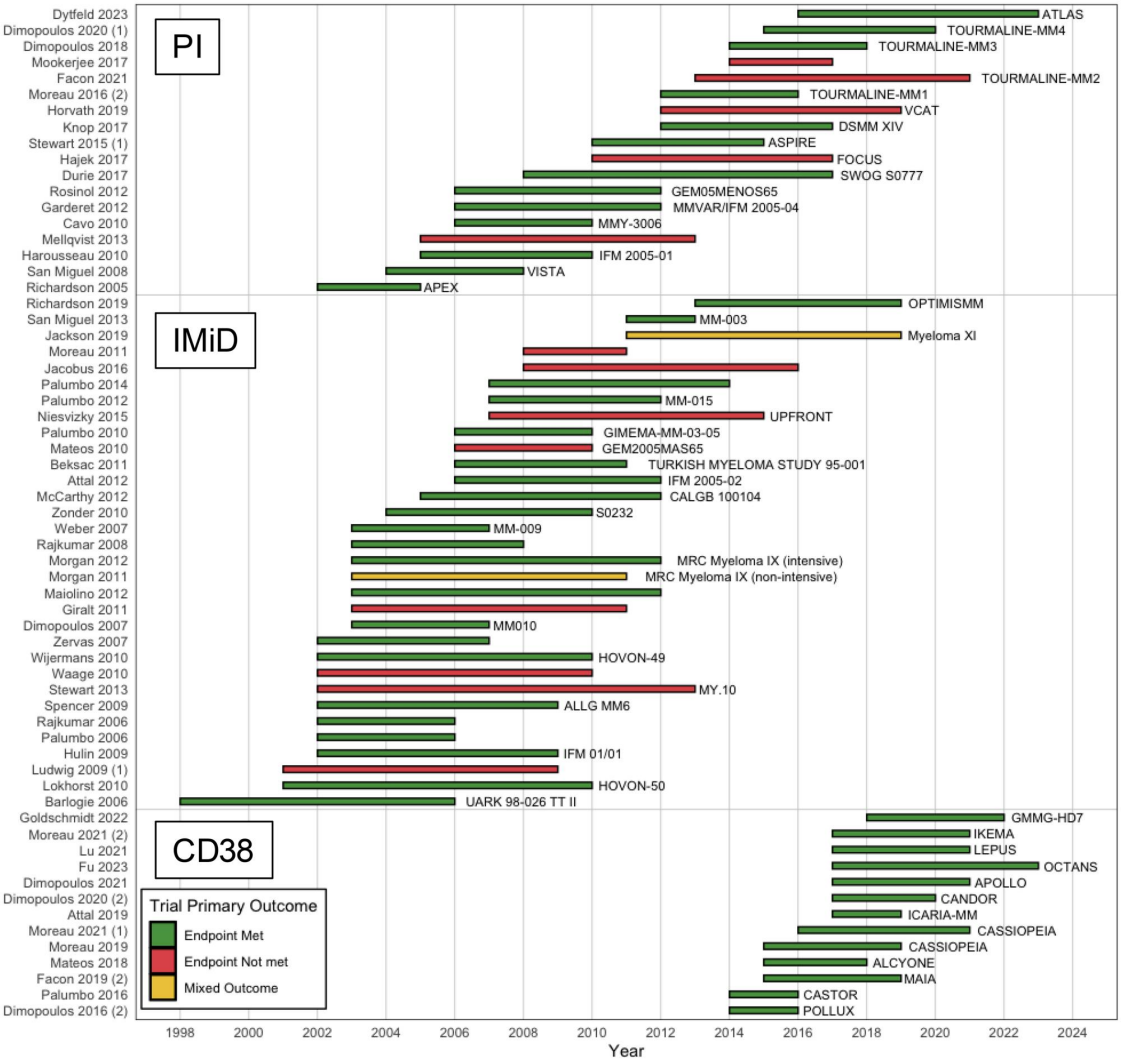

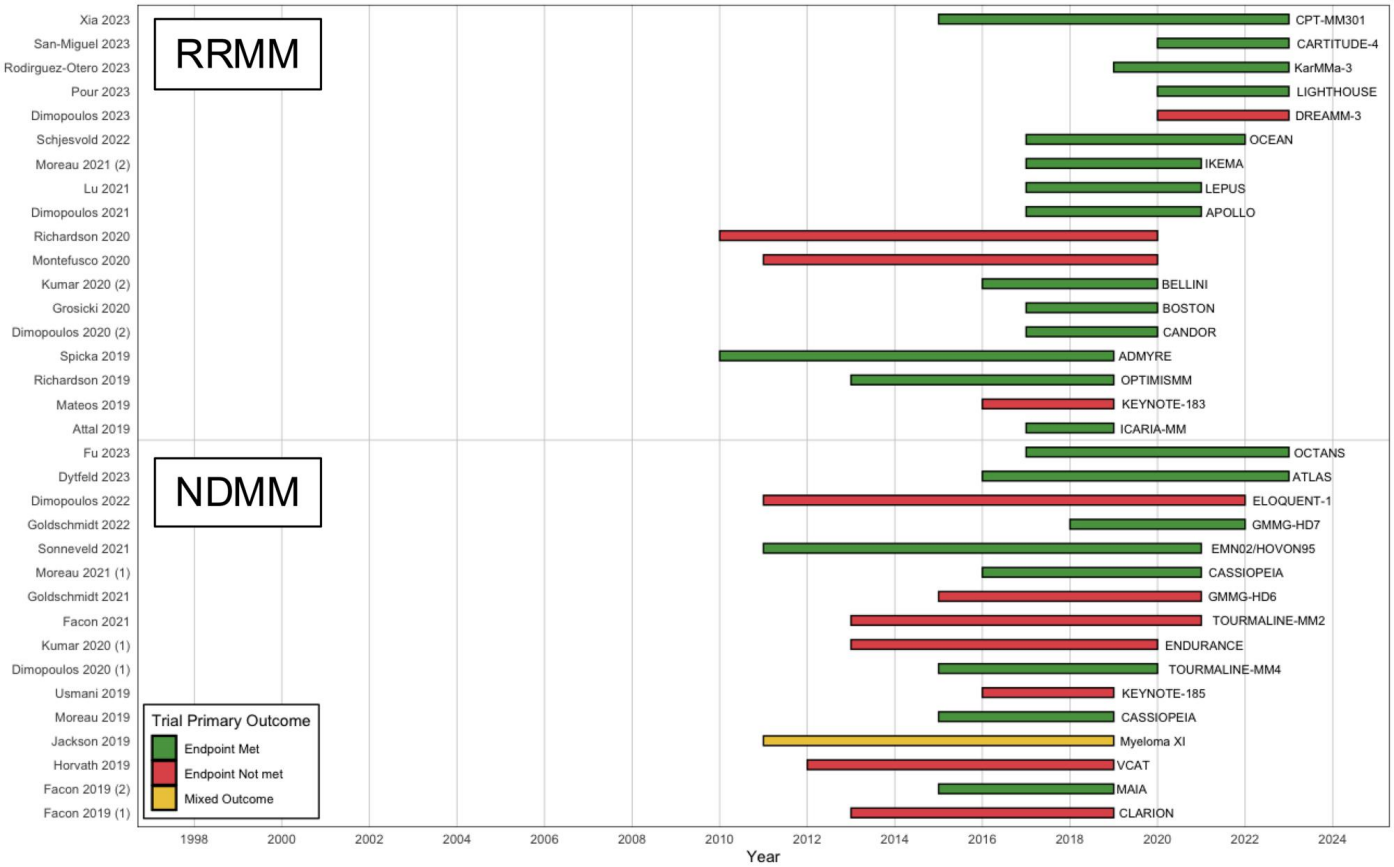
(**A**) Waterfall plot of myeloma trials over time, categorized by class of drugs (proteasome inhibitors [PI], immunomodulatory drug [IMiD], and anti-CD38 monoclonal antibodies [CD38]). Trials with multiple primary outcomes are designated with (1) or (2). Where 1 or more endpoints were met and 1 or more were not met are annotated as having “mixed” results. The *x*-axis indicates the timeline during which the trial was conducted, starting from when the trial started enrolling and ending at when the trial was published. (**B**) Waterfall plot of myeloma trials over the last 5 years, categorized by target population, that is, newly diagnosed vs relapsed/refractory setting (relapsed/refrectory multiple myeloma [RRMM], newly diagnosed multiple myeloma [NDMM]). Trials with multiple primary outcomes are designated with (1) or (2). Where 1 or more endpoints were met and 1 or more were not met are annotated as having “mixed” results. The *x*-axis indicates the timeline during which the trial was conducted, starting from when the trial started enrolling and ending at when the trial was published.

Among comparisons that included proteasome inhibitors, 40 of 61 (66%) met their primary endpoint, whereas for immunomodulatory drugs, 59 of 99 (60%) met their primary endpoint, and for anti-CD38 monoclonal antibodies, all comparisons (15/15) met their primary endpoint. Figure 2, A shows a waterfall plot of myeloma studies over time, showing studies that isolated the effects of each class of drugs: proteasome inhibitors, immunomodulatory drugs, or anti-CD38 monoclonal antibodies. A total of 55 of 103 (53%) comparisons in the newly diagnosed setting and 29 of 42 (69%) comparisons in the relapsed/refractory setting met their endpoints. Figure 2, B shows a waterfall plot of myeloma studies over time, categorized as newly diagnosed vs relapsed/refractory settings.

### Pivotal studies leading to FDA regulatory approval

Of the 84 comparisons that met their endpoints, approximately one-third (27/84, 35%) led directly to FDA approval. Studies leading to regulatory approval enrolled 614 patients (range = 419-722). Five (19%) were substitution studies, whereas 22 (81%) were add-on studies. Overall survival was not the primary endpoint in any study. Progression-free survival was the primary endpoint in 20 (74%) pivotal studies, time-to-progression in 7 (26%), and response in 2 (7%) studies. An OS advantage was demonstrated in 14 (52%) studies that led to approval. Regarding study design, 7 (26%) studies were double blind, 1 (4%) was single blind (to outcome assessors), and the remainder (70%) were open label.

### Univariate analysis

Univariate regression analysis identified several factors predicting whether a study met its primary endpoint (Table 2). These included funding (if they reported receiving funding from the pharmaceutical industry vs not), blinding, primary outcome (OS vs progression outcomes), median age of study participants, inclusion of anti-CD38 monoclonal antibodies, and study participants’ eligibility for transplant. Important factors that did not predict likelihood of meeting a trial’s endpoint included the era when a study was conducted (treating the year of publication as a continuous variable), and whether the study was in the newly diagnosed or relapsed/refractory setting. The study design (head-to-head vs add-on comparison) was not a statistically significant predictor; however, as it was a key question in our study, it was included in further analyses.

**Table 2.** Univariate and multivariate logistic regression analysis of factors predicting whether a trial met its endpoint.

Variables	Univariate models		Multivariate model	
	OR (95% CI)	*P*	OR (95% CI)	*P*
Head-to-head vs add-on	0.60 (0.29 to 1.24)	.170	0.59 (0.22 to 1.62)	.300
Blinded vs open label	5.27 (1.43 to 19.4)	**.013**	7.46 (0.94 to 58.9)	.057
Primary outcome: OS vs PFS/progression	0.09 (0.02 to 0.31)	**<.001**	0.06 (0.01 to 0.23)	**<.001**
Primary outcome: Response/MRD vs PFS/progression	0.73 (0.30 to 1.80)	.490	1.28 (0.38 to 4.31)	.690
Sample size, 2-fold change	1.36 (0.87 to 2.12)	.170	1.60 (0.92 to 2.79)	.100
Setting: Relapsed vs newly diagnosed	1.95 (0.81 to 4.73)	.140	2.24 (0.68 to 7.41)	.180
Setting: Maintenance vs newly diagnosed	1.01 (0.43 to 2.40)	.980	1.64 (0.31 to 8.76)	.560
Industry funded vs other funding	3.03 (1.27 to 7.21)	**.013**	2.32 (0.67 to 8.11)	.180
Published after 2010	0.99 (0.45 to 2.18)	.980		
Published after 2015	0.78 (0.37 to 1.63)	.500		
Year of publication as continuous variable	1.00 (0.94 to 1.07)	.970		
Study follow-up	1.00 (0.99 to 1.00)	.300		
Study location: United States vs Europe	0.34 (0.10 to 1.11)	.072	0.38 (0.08 to 1.83)	.220
Study location: Multiple continents vs Europe	2.09 (0.88 to 4.95)	.092	0.73 (0.20 to 2.61)	.620
Study location: Rest of world vs Europe	2.26 (0.51 to 10.10)	.280	3.57 (0.46 to 27.70)	.220
Median age of participants	0.91 (0.85 to 0.97)	**.006**	0.90 (0.83 to 0.98)	**.022**
PI included	1.73 (0.80 to 3.76)	.16		
IMID included	1.24 (0.58 to 2.65)	.58		
Anti-CD38 monoclonal antibody included	∞ (∞ to ∞)	**<.001**		
Transplantation eligible vs ineligible	3.18 (1.05 to 9.63)	**.041**		
Both transplantation eligible/ineligible vs ineligible	1.11 (0.40 to 3.08)	.84		
Transplantation eligibility not reported vs ineligible	2.10 (0.83 to 5.26)	.11		

*P* < .05 are shown in bold.

Abbreviations: CI = confidence interval; OR = odds ratio; OS = overall survival; PFS = progression-free survival.

On univariate analysis, pharmaceutical industry-funded studies were 3 times more likely to meet their primary endpoint than nonindustry-funded studies (OR = 3.03, 95% CI = 1.27 to 7.21; *P *= .013). Double-blind studies or those in which response assessors were blinded to treatment allocation had approximately 5 times greater odds of meeting their primary endpoint compared with open-label studies or those that did not report blinding (OR = 5.27, 95% CI = 1.43 to 19.4; *P *= .013). Choosing OS as the primary outcome compared with other endpoints, such as PFS, was associated with a 91% lower likelihood of meeting the primary endpoint (OR = 0.09, 95% CI = 0.02 to 0.31; *P *< .001). A higher median age of study participants was also associated with lower odds of meeting the endpoint, with each year’s decrease in participant age increasing the likelihood of meeting the endpoint by approximately 9% (OR = 0.91, 95% CI = 0.85 to 0.97; *P *= .006). Studies that included only transplant-eligible patients were more than 3 times more likely to meet the endpoint than studies that included only transplant-ineligible patients (OR = 3.18, 95% CI = 1.05 to 9.63; *P *= .041). No statistically significant difference was observed when comparing head-to-head and add-on trials (OR = 0.60, 95% CI = 0.29 to 1.24; *P *= .17). These univariate analysis results should be interpreted as descriptive characteristics and the patterns arising from these were further explored in the prespecified multivariate model.

### Multivariate analysis

**Figure 3.**
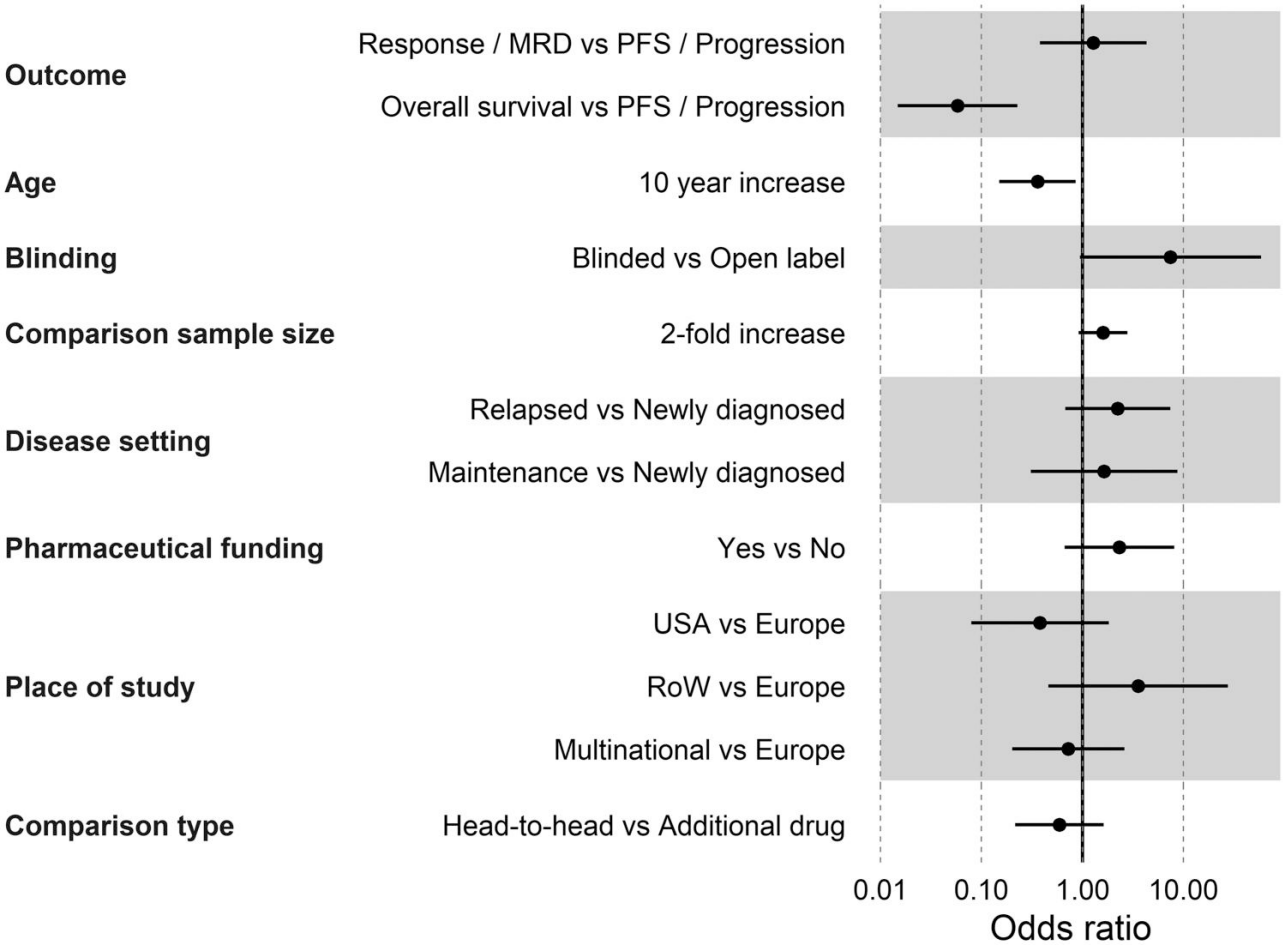
Forest plot demonstrating key variables and their impact on a trial meeting its endpoint.

Eight factors were included in the multivariate analysis: study location, pharmaceutical industry funding, blinding (any blinding vs open label or unknown blinding combined), median age of participants, disease setting (newly diagnosed vs maintenance vs relapsed/refractory), sample size, head-to-head vs add-on comparison, and primary outcome. Of these, 2 factors were independently associated with the likelihood of meeting the primary endpoint. A higher median age of study participants was associated with lower odds of meeting the primary endpoint, with each year’s decrease in participant age increasing the likelihood of meeting the endpoint by approximately 10% (OR = 0.90, 95% CI = 0.83 to 0.98; *P *= .022). Selecting OS as the primary outcome was strongly associated with reduced odds of meeting the endpoint, with a 94% reduction in the likelihood of meeting the endpoint (OR = 0.06; 95% CI = 0.01 to 0.23; *P *< .001) (Figure 3).

### Subgroup analyses

An additional sensitivity analysis separating time-to-event endpoints from response/MRD outcomes was performed. For time-to-event outcomes, in multivariable models, younger median age and progression-based primary endpoints remained independently associated with likelihood of meeting endpoint. For response/MRD endpoints, sample size was associated with likelihood of meeting endpoint, as well as place of study. Other associations were not statistically significant after adjustment. These subgroup analyses are further described in the Supplementary Material.

## Discussion

In this analysis of 22 years of myeloma RCTs, we found that 2 key trial design factors were associated with a higher likelihood of the trial successfully meeting its primary endpoint: including younger patients and using endpoints other than OS as a primary endpoint. Although, historically, head-to-head trial design has been considered riskier than add-on trials, in this regression analysis, we found that they were associated with similar rates of meeting primary endpoints.

These findings extend prior discussions of myeloma clinical trial design by providing a systematic, quantitative assessment of trial and design-level factors associated with success. Previous literature has analyzed specific failures and claimed head-to-head design as a risky trial design that is more likely to be associated with failure, such as in a discussion of the belantamab mafadotin vs pomalidomide/dexamethasone trial.^21^ However, the reasons favoring head-to-head or add-on trial design are complex and often shaped by sponsor strategy, regulatory and clinical context, preexisting evidence, the level of confidence in the experimental treatment, anticipated toxicity, and tolerance for risk. The practical difficulties of demonstrating improved OS in myeloma have been previously discussed,^22-24^ and some surrogate endpoints such as PFS have been considered to be too long to ascertain,^25^ leading to the development of measurable residual disease negativity as a primary endpoint.^26^^,^^27^ This study identifies several key variables in trial design that might be associated with a higher or lower probability of meeting the endpoints. However, ultimately the goal of a trial should be to answer important clinical questions for patients, even if a trial is negative; this should remain the key guiding principle underpinning trial design.

This analysis found that studies enrolling younger patients are associated with higher rates of success, with each year’s decrease in median age increasing the odds by approximately 10%. Although we chose age as a prespecified variable for multivariate analysis, we did not specify a prior hypothesis regarding the interaction of age with outcomes. We hypothesize that reduced attrition, increased tolerance to treatments, and lower competing risks for death from other causes in younger patient populations may have contributed to this finding.^28^ These findings are of immense real-world relevance, as a study with a median age of 60 would have a 2.9-fold higher odds of success compared with a study with a median age of 70. There remains a severe underrepresentation of older patients in clinical trials, especially given that myeloma predominantly affects older patients.^29^ This is exemplified by the exclusion of patients older than age 80 in all 4 RCTs testing quadruplet vs triplet induction regimens^30-33^ or CAR T-cell therapy (NCT04923893) in patients with transplant-ineligible or transplant-deferred NDMM. While these exclusions may reflect safety and feasibility concerns, they risk perpetuating a lack of evidence for the population most affected by the disease. To ensure that clinical trial results are translatable to real-world patients, it is essential that they reflect the age of the patients in the clinic. Randomization stratification, cohort delineation, and further development and implementation of frailty indices may help balance trial efficacy and age inclusiveness.

The selection of OS as the primary endpoint was associated with a decrease in the likelihood of a trial meeting its primary endpoint, and the long follow-up and large sample size required to demonstrate an OS advantage in a disease such as myeloma, where many patients remain stable on treatment for years and multiple lines of treatment are available might explain this finding. The availability of novel therapies postprogression, as well as crossover, may contribute to this. As an example, the confirmatory randomized trial for idecabtagene vicleucel (ide-cel) (KarMMa-3) has not demonstrated an OS benefit, in part due to the inclusion of crossover (ie, subsequent receipt of ide-cel in the control arm),^34^ whereas the confirmatory trial for ciltacabtagene autoleucel (cilta-cel) (CARTITUDE-4) has demonstrated an OS benefit, partly due to low crossover rates.^35^ Although OS may not be a feasible primary efficacy endpoint in many situations, it is a vitally important safety endpoint in all trials, and there are some specific situations where it remains an appropriate primary efficacy endpoint, such as when answering sequencing questions, when evaluating asymptomatic or precursor disease populations, or when evaluating in a very heavily relapsed population where all other options have been exhausted.^13^^,^^24^ It is also critical that trials continue to measure safety endpoints (including OS) long after the primary endpoint has been met to capture the emergence of later safety signals such as secondary malignancies after chimeric antigen receptor T-cell therapies.^35^^,^^36^

These findings underscore the tension between feasibility and clinical relevance in endpoint selection. Evidence shows that frequently used surrogate endpoints only partially predict long-term outcomes. For example, a trial-level meta-analysis found that PFS prolongation accounts for only approximately 40%-60% of the variation in OS,^37^ while achieving a deep response or MRD negativity is not a guarantee of prolonged survival—especially if attained via highly intensive therapy that may compromise patient safety or quality of life. Importantly, no myeloma RCT in our analysis in the past decade has used OS as primary endpoint, and when OS is evaluated, few trials (25 of 145 comparisons in our cohort) demonstrate improved survival. This is in contrast to how patients perceive endpoints, exemplified by a recent survey of patient preferences in myeloma, that highlighted how in many circumstances the majority of patients would not opt for a drug that improves PFS without improving OS.^38^

Recently, several key trials on myeloma have reported negative results. For example, belantamab mafodotin, venetoclax, and melphalan flufenamide each failed in head-to-head trials against pomalidomide plus dexamethasone,^10^^,^^39^ despite some promise in earlier phase trials.^5^^,^^40^ One component contributing to this phenomenon may be that myeloma seems to respond better to combination treatments; hence, the increasing use of quadruplets in frontline treatment is perhaps reflected in the recent positive DREAMM-7 and DREAMM-8 trials of belantamab in triplet combinations.^41^^,^^42^ In addition, some standard care regimens are now performing better than their historical pivotal trials, potentially due to better supportive care and improved performance status and organ function of patients due to more effective prior treatments. However, this might change with the arrival of treatments with potent single-agent activity, such as bispecific antibodies and CAR T-cell therapies. There were no RCTs of these agents during our study time period, and as a result, these newer agents are not accounted for in our analysis.

In multivariate analysis, the use of a head-to-head design was not associated with a reduction in the likelihood of meeting the primary endpoint in our analysis. However, this finding must be interpreted with caution, as multiple factors can influence trial design and outcomes. Head-to-head trials may provide more directly helpful information on how to sequence treatments, as evidenced by the recent 3 vs 3 head-to-head trial that showed belantamab/bortezomib/dexamethasone to be superior to daratumumab/bortezomib/dexamethasone, providing clear guidance for first relapse treatment.^41^ In other hematologic cancers, such as diffuse large B-cell lymphoma and Hodgkin lymphoma, trials have successfully evaluated standards of care through head-to-head design, such as substituting vincristine with polatuzumab vedotin and directly comparing brentuximab with nivolumab in frontline treatment, respectively.^43^ Even if head-to-head trials do not meet their primary endpoint, they can provide clinically meaningful information and allow for direct comparisons of toxicity profiles and quality of life. Such trials in myeloma could help clarify optimal treatment regimens and establish definitive standards of care. Recently successful drugs in myeloma have had many add-on (3 vs 2) trials with different doublet backbones, such as daratumumab and isatuximab.^44-49^ One approach might be to use 1 add-on trial to clearly isolate the effect of the drug to accelerate regulatory approval while recruiting head-to-head trials in earlier lines of therapy to guide clinical practice.

### Limitations

Although we reviewed RCTs in myeloma, given its multiple successful new therapies, these results may not be generalizable to other tumor types. Given the disease-modifying impact of this multitude of therapies on myeloma biology, it is difficult to predict whether our findings will predict the future success of myeloma drug development. Furthermore, as treatment modalities begin to shift toward immunotherapies such as bispecific antibodies and CAR T-cells (the latter typically used as a one-and-done single time point therapy), factors influencing trial success may also shift. Although trials that led to regulatory approval were easy to identify, there is no publicly available mechanism to identify trials that were intended to seek regulatory approval but failed to do so. Publication bias also remains a concern, despite our use of clinical trial registries as a search strategy.

Moreover, it should be acknowledged that this is a regression analysis, and the associations identified should be interpreted with caution, as they are hypothesis generating and do not arise from randomized comparisons. There are inherent limitations to this type of analysis, and several complexities in the trial design may have influenced the results. For example, head-to-head trials often reflect a higher pretest probability that the experimental treatment will outperform the comparator, which could explain why add-on trials were not associated with a higher likelihood of meeting endpoints in this analysis. Power calculations and whether or not power was met in a study were not always provided in the publications and therefore could not be accounted for in our analysis. Finally, design and sponsorship decisions reflect strategic and regulatory considerations that cannot be fully accounted for in this analysis. As an example, a sponsor with a drug that is perceived to be more effective, or a sponsor with greater resources may be more likely to have designed a head-to-head trial rather than an add-on trial, and these factors cannot be fully accounted for. Therefore, these findings should be viewed as hypothesis-generating rather than definitive, and further research is needed to validate these associations.

## Conclusion

As trial design in myeloma and oncology evolves, it remains important to balance the priorities of striving for ever-more-successful new treatment regimens, ensuring that RCTs provide clinicians and patients with actionable, relevant, and translatable information to inform clinical practice. Our analysis identifies key factors that may influence the success of RCTs. We demonstrate that the use of endpoints other than OS and enrollment of a younger patient population are 2 key predictors of a trial meeting its primary endpoint. The wide use of primary surrogate endpoints must be balanced with robust and durable long-term safety and quality-of-life assessments, including collection and reporting of OS, to ensure that novel therapies offer patients a net clinical benefit. Given our results, head-to-head trials may offer an opportunity to optimize the use of combination therapies to maximize patient benefits and inform clinical decision making.

## Supplementary Material

djaf326_Supplementary_Data

## Data Availability

The data are available upon reasonable request to corresponding author.
