# Supplementary material for: Optimizing oncology drug development: systematic review of 22 years of myeloma randomized controlled trials
Source: J Natl Cancer Inst. 2025 Nov 9;118(3):448–58. doi: 10.1093/jnci/djaf326 (PMC13017781; doi:10.1093/jnci/djaf326)
Supplement: djaf326_Supplementary_Data [file djaf326_supplementary_data.zip › Supplementary_Data.docx]

# Table of Contents

## Supplementary Methods .................................................. 2

- Search strategy .................................................. 2
  - PUBMED .................................................. 2
  - COCHRANE (limited to trials) .................................................. 2
  - EMBASE .................................................. 3

## References .................................................. 4

## Tables of Included Studies .................................................. 4

- Table S1: Studies assessing anti-myeloma treatments in newly diagnosed multiple myeloma .................................................. 4
- Table S2: Studies with antimyeloma treatment in Relapsed/Refractory Multiple Myeloma .................................................. 8

## Additional Supplementary Results .................................................. 21

### Regression Modeling .................................................. 21

- Variance inflation .................................................. 21
- Sensitivity analyses without influential observations .................................................. 21
- Visualization of model fit .................................................. 23
  - Effect plots for variables included in the model .................................................. 23
  - Goodness-of-fit plots for variables not included in the model .................................................. 24
- Explore effect of publication year .................................................. 25

### Subgroup Analyses .................................................. 25

- Subgroup analysis by disease setting .................................................. 25
  - Univariate predictors of positive comparisons .................................................. 25
- Subgroup analysis by outcome type .................................................. 28
  - Univariate predictors of positive comparisons .................................................. 28

# Supplementary Methods:

We specified the following independent variables for the multivariable regression analysis: comparison type (add-on or substitution), study design (blinded or open-label), sample size, primary endpoint (progression outcomes [event-free survival, PFS, time to progression or time to treatment failure], OS or response/MRD), funding (pharmaceutical industry funded or non-industry funded); setting (newly diagnosed, maintenance or relapsed), study location (Europe, USA, rest of the world or multicontinental), and participant median age.

Each study was treated as a ‘primary sampling unit’ in complex-survey regression to account for the dependence on the outcomes of multiple comparisons within a study. Study-design related variables were selected for the inclusion in the multivariable model based on substantive interest and not based on univariable pre-screening. For continuous covariates included in the multivariable model, AIC-guided selection was used to select the best-fitting transformation. Missingness in study characteristics was treated as a separate category for categorical characteristics, while for continuous predictors (age), the mean value for non-missing observations was imputed and a missingness indicator was incorporated.

A p-value <0.05 was deemed statistically significant, and all p-values reported are two-tailed. Regression results are presented as Odds Ratios (OR) with 95% Confidence Intervals (CI) and corresponding p-values. Multi-collinearity was assessed using the variance inflation factor (VIF) and a sensitivity analysis removing potential outliers was performed. Statistical analyses were conducted using R Studio (Version 2023.12.1+402).

# Search strategy

We identified RCTs in myeloma from database inception until October 17, 2023, by searching MEDLINE/PubMed, Embase, and the Cochrane Registry of RCTs. We included full text publications and study abstracts. We searched Clinicaltrials.gov for completed studies with reported results. All reports from a given study were consolidated into a single record using NCT number, author names, and study titles. We reviewed the latest FDA label for each myeloma drug to confirm that all studies had been identified using the initial search strategy.

## **PUBMED:**

(((("Multiple Myeloma"[Mesh]) OR "Plasmacytoma"[Mesh]) OR (multiple myeloma OR plasmacytoma OR plasmacytom* OR myelom*))) AND ((randomized controlled trial[pt] OR controlled clinical trial[pt] OR randomized[tiab] OR placebo[tiab] OR clinical trials as topic[mesh:noexp] OR randomly[tiab] OR trial[ti] NOT (animals[mh] NOT humans [mh])))

## **COCHRANE:** (limited to trials)

MeSH descriptor: [Multiple Myeloma] explode all trees

MeSH descriptor: [Plasma Cells] explode all trees

Multiple myeloma:ti,ab,kw

Plasmatocytoma:ti,ab,kw

Plasmatocytom*:ti,ab,kw

Myelom*:ti,ab,kw

## **EMBASE:**

Ab(Multiple Myeloma[Mesh]) OR ab(“multiple myeloma”) OR ab(plasmacytoma*) OR ab(myelom*) OR ab(Plasmacytoma [Mesh])

AND (su.exact.explode(“clinical trial” OR “clinical trial (topic)” OR “clinical trials as topic”) OR qu(“clinical trial”) OR dtype,ti,su,subst(“clinical trial” OR “clinical trials” OR “clin trial” OR “equivalence trial” OR “equivalence trials” OR “multicenter study” OR “multicenter studies” OR “randomized controlled trial” OR “randomized controlled trials”))

AND ((human OR humans OR man OR men OR women OR woman OR patient OR patients OR volunteer OR volunteers OR “homo sapiens” OR Hominidae OR male OR males OR female OR females OR adult OR adults))

# References

1. Horvath N, Spencer A, Kenealy M, et al. Phase 3 study of subcutaneous bortezomib, thalidomide, and prednisolone consolidation after subcutaneous bortezomib-based induction and autologous stem cell transplantation in patients with previously untreated multiple myeloma: the VCAT study. *Leuk Lymphoma*. 2019;60(9):2122-2133. https://www.tandfonline.com/doi/pdf/10.1080/10428194.2019.1579322?needAccess=true

2. Kyle RA, Jacobus S, Friedenberg WR, Slabber CF, Rajkumar SV, Greipp PR. The treatment of multiple myeloma using vincristine, carmustine, melphalan, cyclophosphamide, and prednisone (VBMCP) alternating with high-dose cyclophosphamide and α2β interferon versus VBMCP. *Cancer*. 2009;115(10):2155-2164. doi:10.1002/CNCR.24221

3. Mellqvist UH, Group for the NMS, Gimsing P, et al. Bortezomib consolidation after autologous stem cell transplantation in multiple myeloma: a Nordic Myeloma Study Group randomized phase 3 trial. *Blood*. 2013;121(23):4647-4654. doi:10.1182/BLOOD-2012-11-464503

4. Sonneveld P, Dimopoulos MA, Beksac M, et al. Consolidation and Maintenance in Newly Diagnosed Multiple Myeloma. *Journal of Clinical Oncology*. 2021;39(32):3613-3622. doi:10.1200/JCO.21.01045/ASSET/IMAGES/LARGE/JCO.21.01045TA3.JPEG

5. Spencer A, Prince HM, Roberts AW, et al. Consolidation therapy with low-dose thalidomide and prednisolone prolongs the survival of multiple myeloma patients undergoing a single autologous stem-cell transplantation procedure. *Journal of Clinical Oncology*. 2009;27(11):1788-1793. doi:10.1200/JCO.2008.18.8573/ASSET/IMAGES/ZLJ9990984420003.JPEG

6. Jacobus SJ, Rajkumar S V., Weiss M, et al. Randomized phase III trial of consolidation therapy with bortezomib–lenalidomide–Dexamethasone (VRd) vs bortezomib–dexamethasone (Vd) for patients with multiple myeloma who have completed a dexamethasone based induction regimen. *Blood Cancer J*. 2016;6(7):e448. doi:10.1038/BCJ.2016.55

7. Cavo M, Tacchetti P, Patriarca F, et al. Bortezomib with thalidomide plus dexamethasone compared with thalidomide plus dexamethasone as induction therapy before, and consolidation therapy after, double autologous stem-cell transplantation in newly diagnosed multiple myeloma: A randomised phase 3 study. *The Lancet*. 2010;376(9758):2075-2085. doi:10.1016/S0140-6736(10)61424-9

8. Cook G, Clark RE, Morris TCM, et al. A randomized study (WOS MM1) comparing the oral regime Z-Dex (idarubicin and dexamethasone) with vincristine, adriamycin and dexamethasone as induction therapy for newly diagnosed patients with multiple myeloma. *Br J Haematol*. 2004;126(6):792-798. doi:10.1111/J.1365-2141.2004.05127.X

9. Dimopoulos MA, Pouli A, Zervas K, et al. Prospective randomized comparison of vincristine, doxorubicin and dexamethasone (VAD) administered as intravenous bolus injection and VAD with liposomal doxurubicin as first-line treatment in multiple myeloma. *Annals of Oncology*. 2003;14(7):1039-1044. doi:10.1093/annonc/mdg287

10. Goldschmidt H, Mai EK, Bertsch U, et al. Elotuzumab in Combination with Lenalidomide, Bortezomib, Dexamethasone and Autologous Transplantation for Newly-Diagnosed Multiple Myeloma: Results from the Randomized Phase III GMMG-HD6 Trial. *Blood*. 2021;138(Supplement 1):486-486. doi:10.1182/BLOOD-2021-147323

11. Goldschmidt H, Mai EK, Bertsch U, et al. Addition of isatuximab to lenalidomide, bortezomib, and dexamethasone as induction therapy for newly diagnosed, transplantation-eligible patients with multiple myeloma (GMMG-HD7): part 1 of an open-label, multicentre, randomised, active-controlled, phase 3 trial. *Lancet Haematol*. 2022;9(11):e810-e821. doi:10.1016/S2352-3026(22)00263-0

12. Harousseau JL, Attal M, Avet-Loiseau H, et al. Bortezomib plus dexamethasone is superior to vincristine plus doxorubicin plus dexamethasone as induction treatment prior to autologous stem-cell transplantation in newly diagnosed multiple myeloma: Results of the IFM 2005-01 phase III trial. *Journal of Clinical Oncology*. 2010;28(30):4621-4629. doi:10.1200/JCO.2009.27.9158/ASSET/IMAGES/ZLJ9991003520002.JPEG

13. Hungria VT, Crusoé EQ, Maiolino A, et al. Phase 3 trial of three thalidomide-containing regimens in patients with newly diagnosed multiple myeloma not transplant-eligible. *Ann Hematol*. 2016;95(2):271‐278. https://www.cochranelibrary.com/central/doi/10.1002/central/CN-01169167/full

14. Jackson GH, Pawlyn C, Cairns DA, et al. Carfilzomib, lenalidomide, dexamethasone, and cyclophosphamide (KRdc) as induction therapy for transplant-eligible, newly diagnosed multiple myeloma patients (Myeloma XI+): Interim analysis of an open-label randomised controlled trial. *PLoS Med*. 2021;18(1):e1003454. doi:10.1371/JOURNAL.PMED.1003454

15. Jackson GH, Pawlyn C, Cairns DA, et al. Optimising the value of immunomodulatory drugs during induction and maintenance in transplant ineligible patients with newly diagnosed multiple myeloma: results from Myeloma XI, a multicentre, open-label, randomised, Phase III trial. *Br J Haematol*. 2021;192(5):853-868. doi:10.1111/BJH.16945

16. Knop S, Langer C, Engelhardt MM, et al. Lenalidomide, doxorubicin hydrochloride, and dexamethasone versus bortezomib, lenalidomide, and dexamethasone prior to scheduled stem cell transplant in newly diagnosed myeloma. *Journal of clinical oncology*. 2017;35(15). https://www.cochranelibrary.com/central/doi/10.1002/central/CN-01718733/full

17. Kumar L, Mookerjee A, Sharma A, Gupta R, Sharma OD, Srinivas V. Low dose dexamethasone plus lenalidomide (Len-dexa) versus thalidomide (Thal-dexa) as induction therapy for newly diagnosed multiple myeloma: A Phase III, randomized study. *Clin Lymphoma Myeloma Leuk*. 2015;15:e146. doi:10.1016/j.clml.2015.07.349

18. Kumar SK, Jacobus SJ, Cohen AD, et al. Carfilzomib or bortezomib in combination with lenalidomide and dexamethasone for patients with newly diagnosed multiple myeloma without intention for immediate autologous stem-cell transplantation (ENDURANCE): a multicentre, open-label, phase 3, randomised, controlled trial. *Lancet Oncol*. 2020;21(10):1317-1330. doi:10.1016/S1470-2045(20)30452-6

19. Lokhorst HM, Van Der Holt B, Zweegman S, et al. A randomized phase 3 study on the effect of thalidomide combined with adriamycin, dexamethasone, and high-dose melphalan, followed by thalidomide maintenance in patients with multiple myeloma. *Blood*. 2010;115(6):1113-1120. doi:10.1182/BLOOD-2009-05-222539

20. Ludwig H, Hajek R, Tóthová E, et al. Thalidomide-dexamethasone compared with melphalan-prednisolone in elderly patients with multiple myeloma. *Blood*. 2009;113(15):3435-3442. doi:10.1182/BLOOD-2008-07-169565

21. Ludwig H, Adam Z, Tóthová E, et al. Thalidomide-Interferon Vs. Interferon Maintenance Therapy After Thal-Dex Vs. MP Induction Therapy in Elderly Patients with Multiple Myeloma. *Blood*. 2009;114(22):2891-2891. doi:10.1182/BLOOD.V114.22.2891.2891

22. Magarotto V, Bringhen S, Offidani M, et al. Triplet vs doublet lenalidomide-containing regimens for the treatment of elderly patients with newly diagnosed multiple myeloma. *Blood*. 2016;127(9):1102-1108. https://core.ac.uk/download/84481361.pdf

23. Mai EK, Bertsch U, Dürig J, et al. Phase III trial of bortezomib, cyclophosphamide and dexamethasone (VCD) versus bortezomib, doxorubicin and dexamethasone (PAd) in newly diagnosed myeloma. *Leukemia 2015 29:8*. 2015;29(8):1721-1729. doi:10.1038/leu.2015.80

24. Mateos MV, Oriol A, Martínez-López J, et al. Bortezomib, melphalan, and prednisone versus bortezomib, thalidomide, and prednisone as induction therapy followed by maintenance treatment with bortezomib and thalidomide versus bortezomib and prednisone in elderly patients with untreated multiple myeloma: A randomised trial. *Lancet Oncol*. 2010;11(10):934-941. doi:10.1016/S1470-2045(10)70187-X

25. Mateos MV, Dimopoulos MA, Cavo M, et al. Daratumumab plus Bortezomib, Melphalan, and Prednisone for Untreated Myeloma. *N Engl J Med*. 2018;378(6):518-528. doi:10.1056/NEJMOA1714678

26. Mookerjee A, Gupta R, Jasrotia S, et al. Bortezomib, Lenalidomide and Low-Dose Dexamethasone (VRD) Versus Lenalidomide and Low-Dose Dexamethasone (Ld) for Newly-Diagnosed Multiple Myeloma- a Randomized Phase III Study. *Blood*. 2017;130(Supplement 1):906-906. doi:10.1182/BLOOD.V130.SUPPL_1.906.906

27. Moreau P, Avet-Loiseau H, Facon T, Attal M, Tiab M, Hulin C. Bortezomib plus dexamethasone versus reduced-dose bortezomib, thalidomide plus dexamethasone as induction treatment before autologous stem cell transplantation in newly diagnosed multiple myeloma. *Blood*. 2011;118(22):5752‐5758.

28. Moreau P, Hulin C, Macro M, et al. VTD is superior to VCD prior to intensive therapy in multiple myeloma: results of the prospective IFM2013-04 trial. *Blood*. 2016;127(21):2569-2574. doi:10.1182/BLOOD-2016-01-693580

29. Moreau P, Attal M, Hulin C, et al. Bortezomib, thalidomide, and dexamethasone with or without daratumumab before and after autologous stem-cell transplantation for newly diagnosed multiple myeloma (CASSIOPEIA): a randomised, open-label, phase 3 study. *The Lancet*. 2019;394(10192):29-38. doi:10.1016/S0140-6736(19)31240-1

30. Morgan GJ, Davies FE, Gregory WM, et al. Cyclophosphamide, thalidomide, and dexamethasone (CTD) as initial therapy for patients with multiple myeloma unsuitable for autologous transplantation. *Blood*. 2011;118(5):1231. doi:10.1182/BLOOD-2011-02-338665

31. Morgan GJ, Davies FE, Gregory WM, et al. Cyclophosphamide, thalidomide, and dexamethasone as induction therapy for newly diagnosed multiple myeloma patients destined for autologous stem-cell transplantation: MRC Myeloma IX randomized trial results. *Haematologica*. 2012;97(3):442-450. doi:10.3324/haematol.2011.043372

32. Niesvizky R, Flinn IW, Rifkin R, et al. Community-based phase IIIB trial of three UPFRONT bortezomib-based myeloma regimens. *Journal of Clinical Oncology*. 2015;33(33):3921-3929. doi:10.1200/JCO.2014.58.7618/SUPPL_FILE/PROTOCOL_2014.587618.PDF

33. Palumbo A, Bringhen S, Rossi D, et al. Bortezomib-melphalan-prednisone-thalidomide followed by maintenance with bortezomib-thalidomide compared with bortezomib-melphalan-prednisone for initial treatment of multiple myeloma: A randomized controlled trial. *Journal of Clinical Oncology*. 2010;28(34):5101-5109. doi:10.1200/JCO.2010.29.8216/ASSET/IMAGES/ZLJ9991006150003.JPEG

34. Pönisch W, Mitrou PS, Merkle K, et al. Treatment of Bendamustine and Prednisone in patients with newly diagnosed multiple myeloma results in superior complete response rate, prolonged time to treatment failure and improved quality of life compared to treatment with Melphalan and Prednisone - A randomized phase III study of the East German Study Group of Hematology and Oncology (OSHO). *J Cancer Res Clin Oncol*. 2006;132(4):205-212. doi:10.1007/S00432-005-0074-4/METRICS

35. Rajkumar SV, Blood E, Vesole D, Fonseca R, Greipp PR. Phase III clinical trial of thalidomide plus dexamethasone compared with dexamethasone alone in newly diagnosed multiple myeloma: A clinical trial coordinated by the eastern cooperative oncology group. *Journal of Clinical Oncology*. 2006;24(3):431-436. doi:10.1200/JCO.2005.03.0221/ASSET/IMAGES/ZLJ0030631820001.JPEG

36. Rajkumar SV, Rosiñol L, Hussein M, et al. Multicenter, randomized, double-blind, placebo-controlled study of thalidomide plus dexamethasone compared with dexamethasone as initial therapy for newly diagnosed multiple myeloma. *Journal of Clinical Oncology*. 2008;26(13):2171-2177. doi:10.1200/JCO.2007.14.1853/ASSET/IMAGES/ZLJ0130871280003.JPEG

37. Rifkin RM, Gregory SA, Mohrbacher A, et al. Pegylated liposomal doxorubicin, vincristine, and dexamethasone provide significant reduction in toxicity compared with doxorubicin, vincristine, and dexamethasone in patients with newly diagnosed multiple myeloma. *Cancer*. 2006;106(4):848-858. doi:10.1002/CNCR.21662

38. Rosiñol L, group on behalf of the P para el E y la T de las HME de M (PETHEMA/GEM), Oriol A, et al. Superiority of bortezomib, thalidomide, and dexamethasone (VTD) as induction pretransplantation therapy in multiple myeloma: a randomized phase 3 PETHEMA/GEM study. *Blood*. 2012;120(8):1589-1596. doi:10.1182/BLOOD-2012-02-408922

39. Shustik C, Belch A, Robinson S, et al. A randomised comparison of melphalan with prednisone or dexamethasone as induction therapy and dexamethasone or observation as maintenance therapy in multiple myeloma: NCIC CTG MY.7. *Br J Haematol*. 2007;136(2):203-211. doi:10.1111/J.1365-2141.2006.06405.X

40. Straka C, Liebisch P, Salwender H, et al. Autotransplant with and without induction chemotherapy in older multiple myeloma patients: long-term outcome of a randomized trial. *Haematologica*. 2016;101(11):1398. doi:10.3324/HAEMATOL.2016.151860

41. Usmani SZ, Schjesvold F, Oriol A, et al. Pembrolizumab plus lenalidomide and dexamethasone for patients with treatment-naive multiple myeloma (KEYNOTE-185): a randomised, open-label, phase 3 trial. *Lancet Haematol*. 2019;6(9):e448-e458. doi:10.1016/S2352-3026(19)30109-7

42. Zervas K, Mihou D, Katodritou E, et al. VAD-doxil versus VAD-doxil plus thalidomide as initial treatment for multiple myeloma: Results of a multicenter randomized trial of the Greek myeloma study group. *Annals of Oncology*. 2007;18(8):1369-1375. doi:10.1093/annonc/mdm178

43. Jackson GH, Davies FE, Pawlyn C, et al. Response-adapted intensification with cyclophosphamide, bortezomib, and dexamethasone versus no intensification in patients with newly diagnosed multiple myeloma (Myeloma XI): a multicentre, open-label, randomised, phase 3 trial. *Lancet Haematol*. 2019;6(12):e616. doi:10.1016/S2352-3026(19)30167-X

44. Attal M, Lauwers-Cances V, Marit G, et al. Lenalidomide maintenance after stem-cell transplantation for multiple myeloma. *N Engl J Med*. 2012;366(19):1782-1791. doi:10.1056/NEJMOA1114138

45. Barlogie B, Kyle RA, Anderson KC, et al. Standard chemotherapy compared with high-dose chemoradiotherapy for multiple myeloma: Final results of phase III US intergroup trial S9321. *Journal of Clinical Oncology*. 2006;24(6):929-936. doi:10.1200/JCO.2005.04.5807/ASSET/IMAGES/ZLJ0060633090005.JPEG

46. Bringhen S, Offidani M, Musto P, et al. Long Term Outcome of Lenalidomide-Dexamethasone (Rd) Vs Melphalan-Lenalidomide-Prednisone (MPR) Vs Cyclophosphamide-Prednisone-Lenalidomide (CPR) As Induction Followed By Lenalidomide-Prednisone (RP) Vs Lenalidomide (R) As Maintenance in a Community-Based Newly Diagnosed Myeloma Population: Updated Analysis of EMN01 Phase III Study. *Blood*. 2017;130(Supplement 1):901-901. doi:10.1182/BLOOD.V130.SUPPL_1.901.901

47. Dimopoulos MA, Gay F, Schjesvold F, et al. Oral ixazomib maintenance following autologous stem cell transplantation (TOURMALINE-MM3): a double-blind, randomised, placebo-controlled phase 3 trial. *The Lancet*. 2019;393(10168):253-264. doi:10.1016/S0140-6736(18)33003-4

48. Dimopoulos MA, Špička I, Quach H, et al. Ixazomib as Postinduction Maintenance for Patients With Newly Diagnosed Multiple Myeloma Not Undergoing Autologous Stem Cell Transplantation: The Phase III TOURMALINE-MM4 Trial. *Journal of Clinical Oncology*. 2020;38(34):4030. doi:10.1200/JCO.20.02060

49. Dytfeld D, Wróbel T, Jamroziak K, et al. Carfilzomib, lenalidomide, and dexamethasone or lenalidomide alone as maintenance therapy after autologous stem-cell transplantation in patients with multiple myeloma (ATLAS): interim analysis of a randomised, open-label, phase 3 trial. *Lancet Oncol*. 2023;24(2):139. doi:10.1016/S1470-2045(22)00738-0

50. Gay F, Oliva S, Petrucci MT, et al. Chemotherapy plus lenalidomide versus autologous transplantation, followed by lenalidomide plus prednisone versus lenalidomide maintenance, in patients with multiple myeloma: A randomised, multicentre, phase 3 trial. *Lancet Oncol*. 2015;16(16):1617-1629. doi:10.1016/S1470-2045(15)00389-7

51. Giralt S, Costa LJ, Maloney D, et al. Tandem Autologous-Autologous versus Autologous-Allogeneic Hematopoietic Stem Cell Transplant for Patients with Multiple Myeloma: Long-Term Follow-Up Results from the Blood and Marrow Transplant Clinical Trials Network 0102 Trial. *Biol Blood Marrow Transplant*. Published online 2019.

52. Jackson GH, Davies FE, Pawlyn C, et al. Lenalidomide maintenance versus observation for patients with newly diagnosed multiple myeloma (Myeloma XI): a multicentre, open-label, randomised, phase 3 trial. *Lancet Oncol*. 2019;20(1):57-73. https://www.ncbi.nlm.nih.gov/pmc/articles/PMC6318225/pdf/main.pdf

53. Mateos MV, Oriol A, Martínez-López J, et al. Maintenance therapy with bortezomib plus thalidomide or bortezomib plus prednisone in elderly multiple myeloma patients included in the GEM2005MAS65 trial. *Blood*. 2012;120(13):2581-2588. doi:10.1182/BLOOD-2012-05-427815

54. Moreau P, Hulin C, Perrot A, et al. Maintenance with daratumumab or observation following treatment with bortezomib, thalidomide, and dexamethasone with or without daratumumab and autologous stem-cell transplant in patients with newly diagnosed multiple myeloma (CASSIOPEIA): an open-label, randomised, phase 3 trial. *Lancet Oncol*. 2021;22(10):1378-1390. doi:10.1016/S1470-2045(21)00428-9

55. Morgan GJ, Gregory WM, Davies FE, et al. The role of maintenance thalidomide therapy in multiple myeloma: MRC Myeloma IX results and meta-analysis. *Blood*. 2012;119(1):7-15.

56. Palumbo A, Cavallo F, Gay F, et al. Autologous Transplantation and Maintenance Therapy in Multiple Myeloma. *New England Journal of Medicine*. 2014;371(10):895-905. doi:10.1056/NEJMOA1402888/SUPPL_FILE/NEJMOA1402888_DISCLOSURES.PDF

57. Rosiñol L, Oriol A, Teruel AI, et al. Bortezomib and thalidomide maintenance after stem cell transplantation for multiple myeloma: a PETHEMA/GEM trial. *Leukemia 2017 31:9*. 2017;31(9):1922-1927. doi:10.1038/leu.2017.35

58. Schaar CG, Kluin-Nelemans HC, Te Marvelde C, et al. Interferon-alpha as maintenance therapy in patients with multiple myeloma. *Ann Oncol*. 2005;16(4):634-639.

59. Shustik C, Belch A, Robinson S, et al. Dexamethasone (dex) maintenance versus observation (obs) in patients with previously untreated multiple myeloma: a National Cancer Institute Of Canada Clinical Trials Group Study: mY.7. *Journal of clinical oncology : ASCO annual meeting proceedings*. 2004;22(14S):558. https://www.cochranelibrary.com/central/doi/10.1002/central/CN-00580703/full

60. Barlogie B, Tricot G, Anaissie E, et al. Thalidomide and Hematopoietic-Cell Transplantation for Multiple Myeloma. *New England Journal of Medicine*. 2006;354(10):1021-1030. doi:10.1056/NEJMOA053583/SUPPL_FILE/NEJM_BARLOGIE_1021SA1.PDF

61. Beksac M, Haznedar R, Firatli-Tuglular T, et al. Addition of thalidomide to oral melphalan/prednisone in patients with multiple myeloma not eligible for transplantation: results of a randomized trial from the Turkish Myeloma Study Group. *Eur J Haematol*. 2011;86(1):16-22. doi:10.1111/J.1600-0609.2010.01524.X

62. Benboubker L, Dimopoulos MA, Dispenzieri A, et al. Lenalidomide and Dexamethasone in Transplant-Ineligible Patients with Myeloma. *New England Journal of Medicine*. 2014;371(10):906-917. doi:10.1056/NEJMOA1402551/SUPPL_FILE/NEJMOA1402551_DISCLOSURES.PDF

63. A Study of Thalidomide Plus Dexamethasone (Thal-Dex) Versus DOXIL plusThalidomide Plus Dexamethasone (DOXIL -Thal-Dex) in Patients With Newly Diagnosed Multiple Myeloma. https://ClinicalTrials.gov/show/NCT00097981

64. Dimopoulos MA, Richardson PG, Bahlis NJ, et al. Addition of elotuzumab to lenalidomide and dexamethasone for patients with newly diagnosed, transplantation ineligible multiple myeloma (ELOQUENT-1): an open-label, multicentre, randomised, phase 3 trial. *Lancet Haematol*. 2022;9(6):e403-e414. doi:10.1016/S2352-3026(22)00103-X

65. Durie BGM, Hoering A, Abidi MH, et al. Bortezomib with lenalidomide and dexamethasone versus lenalidomide and dexamethasone alone in patients with newly diagnosed myeloma without intent for immediate autologous stem-cell transplant (SWOG S0777): a randomised, open-label, phase 3 trial HHS Public Access. *Lancet*. 2017;389:519-527. doi:10.1016/S0140-6736(16)31594-X

66. Facon T, Lee JH, Moreau P, et al. Carfilzomib or bortezomib with melphalan-prednisone for transplant-ineligible patients with newly diagnosed multiple myeloma. *Blood*. 2019;133(18):1953-1963. doi:10.1182/BLOOD-2018-09-874396

67. Facon T, Kumar S, Plesner T, et al. Daratumumab plus Lenalidomide and Dexamethasone for Untreated Myeloma. *N Engl J Med*. 2019;380(22):2104-2115. doi:10.1056/NEJMOA1817249

68. Facon T, Venner CP, Bahlis NJ, et al. Oral ixazomib, lenalidomide, and dexamethasone for transplant-ineligible patients with newly diagnosed multiple myeloma. *Blood*. 2021;137(26):3616-3628. doi:10.1182/BLOOD.2020008787

69. Fu W, Bang SM, Huang H, et al. Bortezomib, Melphalan, and Prednisone With or Without Daratumumab in Transplant-ineligible Asian Patients With Newly Diagnosed Multiple Myeloma: The Phase 3 OCTANS Study. *Clin Lymphoma Myeloma Leuk*. 2023;23(6):446-455.e4. doi:10.1016/j.clml.2023.02.009

70. Hulin C, Facon T, Rodon P, et al. Efficacy of melphalan and prednisone plus thalidomide in patients older than 75 years with newly diagnosed multiple myeloma: IFM 01/01 trial. *Journal of Clinical Oncology*. 2009;27(22):3664-3670. doi:10.1200/JCO.2008.21.0948/ASSET/IMAGES/ZLJ9990986940002.JPEG

71. Palumbo A, Bringhen S, Caravita T, et al. Oral melphalan and prednisone chemotherapy plus thalidomide compared with melphalan and prednisone alone in elderly patients with multiple myeloma: randomised controlled trial. *Lancet*. 2006;367(9513):825-831. doi:10.1016/S0140-6736(06)68338-4

72. Palumbo A, Hajek R, Delforge M, et al. Continuous Lenalidomide Treatment for Newly Diagnosed Multiple Myeloma. *New England Journal of Medicine*. 2012;366(19):1759-1769. doi:10.1056/NEJMOA1112704/SUPPL_FILE/NEJMOA1112704_DISCLOSURES.PDF

73. San Miguel JF, Schlag R, Khuageva NK, et al. Bortezomib plus Melphalan and Prednisone for Initial Treatment of Multiple Myeloma. *New England Journal of Medicine*. 2008;359(9):906-917. doi:10.1056/NEJMOA0801479/SUPPL_FILE/NEJM_SAN_MIGUEL_906SA1.PDF

74. Sonneveld P, Schmidt-Wolf IGH, Van Der Holt B, et al. Bortezomib induction and maintenance treatment in patients with newly diagnosed multiple myeloma: Results of the randomized phase III HOVON-65/ GMMG-HD4 trial. *Journal of Clinical Oncology*. 2012;30(24):2946-2955. doi:10.1200/JCO.2011.39.6820

75. Stewart AK, Jacobus S, Fonseca R, et al. Melphalan, prednisone, and thalidomide vs melphalan, prednisone, and lenalidomide (ECOG E1A06) in untreated multiple myeloma. *Blood*. 2015;126(11):1294. doi:10.1182/BLOOD-2014-12-613927

76. Takenaka T, Itoh K, Suzuki T, et al. Phase III Study of Ranimustine, Cyclophosphamide, Vincristine, Melphalan, and Prednisolone (MCNU-COP/MP) versus Modified COP/MP in Multiple Myeloma: A Japan Clinical Oncology Group Study, JCOG 9301. *Int J Hematol*. 2004;79(2):165-173. doi:10.1532/IJH97.03115/METRICS

77. Waage A, Gimsing P, Fayers P, et al. Melphalan and prednisone plus thalidomide or placebo in elderly patients with multiple myeloma. *Blood*. 2010;116(9):1405-1412. doi:10.1182/BLOOD-2009-08-237974

78. Wijermans P, Schaafsma M, Termorshuizen F, et al. Phase III study of the value of thalidomide added to melphalan plus prednisone in elderly patients with newly diagnosed multiple myeloma: The HOVON 49 study. *Journal of Clinical Oncology*. 2010;28(19):3160-3166. doi:10.1200/JCO.2009.26.1610/ASSET/IMAGES/ZLJ9991001690003.JPEG

79. Zonder JA, Crowley J, Hussein MA, et al. Lenalidomide and high-dose dexamethasone compared with dexamethasone as initial therapy for multiple myeloma: a randomized Southwest Oncology Group trial (S0232). *Blood*. 2010;116(26):5838-5841. doi:10.1182/BLOOD-2010-08-303487

80. Zweegman S, Van Der Holt B, Mellqvist UH, et al. Melphalan, prednisone, and lenalidomide versus melphalan, prednisone, and thalidomide in untreated multiple myeloma. *Blood*. 2016;127(9):1109-1116. doi:10.1182/BLOOD-2015-11-679415

81. Maiolino A, Hungria VTM, Garnica M, et al. Thalidomide plus dexamethasone as a maintenance therapy after autologous hematopoietic stem cell transplantation improves progression-free survival in multiple myeloma. *Am J Hematol*. 2012;87(10):948-952. doi:10.1002/AJH.23274

82. Stewart AK, Trudel S, Bahlis NJ, et al. A randomized phase 3 trial of thalidomide and prednisone as maintenance therapy after ASCT in patients with MM with a quality-of-life assessment: the National Cancer Institute of Canada Clinicals Trials Group Myeloma 10 Trial. *Blood*. 2013;121(9):1517-1523. doi:10.1182/BLOOD-2012-09-451872

83. Offidani M, Corvatta L, Polloni C, et al. Thalidomide-dexamethasone versus interferon-alpha-dexamethasone as maintenance treatment after ThaDD induction for multiple myeloma: a prospective, multicentre, randomised study. *Br J Haematol*. 2009;144(5):653-659. doi:10.1111/J.1365-2141.2008.07495.X

84. Mccarthy PL, Owzar K, Hofmeister CC, et al. Lenalidomide after Stem-Cell Transplantation for Multiple Myeloma. *N Engl J Med*. 2012;366:1770-1781.

85. Montefusco V, Corso A, Galli M, et al. Bortezomib, cyclophosphamide, dexamethasone versus lenalidomide, cyclophosphamide, dexamethasone in multiple myeloma patients at first relapse. *Br J Haematol*. 2020;188(6):907-917. doi:10.1111/BJH.16287

86. Attal M, Richardson PG, Rajkumar S V, et al. Isatuximab plus pomalidomide and low-dose dexamethasone versus pomalidomide and low-dose dexamethasone in patients with relapsed and refractory multiple myeloma (ICARIA-MM): a randomised, multicentre, open-label, phase 3 study. *Lancet*. 2019;394(10214):2096-2107.

87. Chanan-Khan A, Niesvizky R, Hohl RJ, et al. Phase III randomised study of dexamethasone with or without oblimersen sodium for patients with advanced multiple myeloma. *Leuk Lymphoma*. 2009;50(4):559-565. doi:10.1080/10428190902748971

88. Dimopoulos M, Spencer A, Attal M, et al. Lenalidomide plus Dexamethasone for Relapsed or Refractory Multiple Myeloma. *New England Journal of Medicine*. 2007;357(21):2123-2132. doi:10.1056/NEJMOA070594

89. Dimopoulos M, Siegel DS, Lonial S, et al. Vorinostat or placebo in combination with bortezomib in patients with multiple myeloma (VANTAGE 088): A multicentre, randomised, double-blind study. *Lancet Oncol*. 2013;14(11):1129-1140. doi:10.1016/S1470-2045(13)70398-X

90. Dimopoulos MA, Moreau P, Palumbo A, et al. Carfilzomib and dexamethasone versus bortezomib and dexamethasone for patients with relapsed or refractory multiple myeloma (ENDEAVOR): And randomised, phase 3, open-label, multicentre study. *Lancet Oncol*. 2016;17(1):27-38. doi:10.1016/S1470-2045(15)00464-7

91. Dimopoulos MA, Oriol A, Nahi H, et al. Daratumumab, Lenalidomide, and Dexamethasone for Multiple Myeloma. *New England Journal of Medicine*. 2016;375(14):1319-1331. doi:10.1056/NEJMOA1607751/SUPPL_FILE/NEJMOA1607751_DISCLOSURES.PDF

92. Dimopoulos M, Quach H, Mateos MV, et al. Carfilzomib, dexamethasone, and daratumumab versus carfilzomib and dexamethasone for patients with relapsed or refractory multiple myeloma (CANDOR): results from a randomised, multicentre, open-label, phase 3 study. *The Lancet*. 2020;396(10245):186-197. doi:10.1016/S0140-6736(20)30734-0

93. Dimopoulos MA, Terpos E, Boccadoro M, et al. Daratumumab plus pomalidomide and dexamethasone versus pomalidomide and dexamethasone alone in previously treated multiple myeloma (APOLLO): an open-label, randomised, phase 3 trial. *Lancet Oncol*. 2021;22(6):801-812. doi:10.1016/S1470-2045(21)00128-5

94. Dimopoulos MA, Hungria VTM, Radinoff A, et al. Efficacy and safety of single-agent belantamab mafodotin versus pomalidomide plus low-dose dexamethasone in patients with relapsed or refractory multiple myeloma (DREAMM-3): a phase 3, open-label, randomised study. *Lancet Haematol*. 2023;10(10):e801-e812. doi:10.1016/S2352-3026(23)00243-0

95. Friedenberg WR, Rue M, Blood EA, et al. Phase III study of PSC-833 (valspodar) in combination with vincristine, doxorubicin, and dexamethasone (valspodar/VAD) versus VAD alone in patients with recurring or refractory multiple myeloma (E1A95). *Cancer*. 2006;106(4):830-838. doi:10.1002/CNCR.21666

96. Garderet L, Iacobelli S, Moreau P, et al. Superiority of the triple combination of bortezomib-thalidomide- dexamethasone over the dual combination of thalidomide-dexamethasone in patients with multiple myeloma progressing or relapsing after autologous transplantation: The MMVAR/IFM 2005-04 randomized phase III trial from the chronic leukemia working party of the European Group for blood and marrow transplantation. *Journal of Clinical Oncology*. 2012;30(20):2475-2482. doi:10.1200/JCO.2011.37.4918/SUPPL_FILE/74918_GARDERET_TABLE_2.PDF

97. Grosicki S, Simonova M, Spicka I, et al. Once-per-week selinexor, bortezomib, and dexamethasone versus twice-per-week bortezomib and dexamethasone in patients with multiple myeloma (BOSTON): a randomised, open-label, phase 3 trial. *The Lancet*. 2020;396(10262):1563-1573. doi:10.1016/S0140-6736(20)32292-3

98. Hájek R, Masszi T, Petrucci MT, et al. A randomized phase III study of carfilzomib vs low-dose corticosteroids with optional cyclophosphamide in relapsed and refractory multiple myeloma (FOCUS). *Leukemia*. 2017;31(1):107. doi:10.1038/LEU.2016.176

99. Hjorth M, Hjertner Ø, Knudsen LM, et al. Thalidomide and dexamethasone vs. bortezomib and dexamethasone for melphalan refractory myeloma: a randomized study. *Eur J Haematol*. 2012;88(6):485. doi:10.1111/J.1600-0609.2012.01775.X

100. Kropff M, Vogel M, Bisping G, et al. Bortezomib and low-dose dexamethasone with or without continuous low-dose oral cyclophosphamide for primary refractory or relapsed multiple myeloma: a randomized phase III study. *Ann Hematol*. 2017;96(11):1857-1866. doi:10.1007/S00277-017-3065-Z/METRICS

101. Kumar SK, Harrison SJ, Cavo M, et al. Venetoclax or placebo in combination with bortezomib and dexamethasone in patients with relapsed or refractory multiple myeloma (BELLINI): a randomised, double-blind, multicentre, phase 3 trial. *Lancet Oncol*. 2020;21(12):1630-1642. doi:10.1016/S1470-2045(20)30525-8

102. Lonial S, Dimopoulos M, Palumbo A, et al. Elotuzumab Therapy for Relapsed or Refractory Multiple Myeloma. *New England Journal of Medicine*. 2015;373(7):621-631. doi:10.1056/NEJMOA1505654/SUPPL_FILE/NEJMOA1505654_DISCLOSURES.PDF

103. Lu J, Fu W, Li W, et al. Daratumumab, Bortezomib, and Dexamethasone Versus Bortezomib and Dexamethasone in Chinese Patients with Relapsed or Refractory Multiple Myeloma: Phase 3 LEPUS (MMY3009) Study. *Clin Lymphoma Myeloma Leuk*. 2021;21(9):e699-e709. doi:10.1016/J.CLML.2021.04.012

104. Mateos MV, Blacklock H, Schjesvold F, et al. Pembrolizumab plus pomalidomide and dexamethasone for patients with relapsed or refractory multiple myeloma (KEYNOTE-183): a randomised, open-label, phase 3 trial. *Lancet Haematol*. 2019;6(9):e459-e469. doi:10.1016/S2352-3026(19)30110-3

105. Miguel JS, Weisel K, Moreau P, et al. Pomalidomide plus low-dose dexamethasone versus high-dose dexamethasone alone for patients with relapsed and refractory multiple myeloma (MM-003): A randomised, open-label, phase 3 trial. *Lancet Oncol*. 2013;14(11):1055-1066. doi:10.1016/S1470-2045(13)70380-2

106. Moreau P, Masszi T, Grzasko N, et al. Oral Ixazomib, Lenalidomide, and Dexamethasone for Multiple Myeloma. *N Engl J Med*. 2016;374(17):1621-1634. doi:10.1056/NEJMOA1516282

107. Moreau P, Dimopoulos MA, Mikhael J, et al. Isatuximab, carfilzomib, and dexamethasone in relapsed multiple myeloma (IKEMA): a multicentre, open-label, randomised phase 3 trial. *Lancet*. 2021;397(10292):2361-2371. doi:10.1016/S0140-6736(21)00592-4

108. Orlowski RZ, Nagler A, Sonneveld P, et al. Randomized phase III study of pegylated liposomal doxorubicin plus bortezomib compared with bortezomib alone in relapsed or refractory multiple myeloma: combination therapy improves time to progression. *Journal of clinical oncology*. 2007;25(25):3892-3901. doi:10.1200/JCO.2006.10.5460

109. Palumbo A, Chanan-Khan A, Weisel K, et al. Daratumumab, Bortezomib, and Dexamethasone for Multiple Myeloma. *N Engl J Med*. 2016;375(8):754-766. doi:10.1056/NEJMOA1606038

110. Pour L, Szarejko M, Bila J, et al. Efficacy and safety of melflufen plus daratumumab and dexamethasone in relapsed/refractory multiple myeloma: results from the randomized, open-label, phase III LIGHTHOUSE study. *Haematologica*. 2024;109(3):895-905. doi:10.3324/HAEMATOL.2023.283509

111. Richardson PG, Sonneveld P, Schuster MW, et al. Bortezomib or high-dose dexamethasone for relapsed multiple myeloma. *N Engl J Med*. 2005;352(24):2487-2498. doi:10.1056/NEJMOA043445

112. Richardson PG, Oriol A, Beksac M, et al. Pomalidomide, bortezomib, and dexamethasone for patients with relapsed or refractory multiple myeloma previously treated with lenalidomide (OPTIMISMM): a randomised, open-label, phase 3 trial. *Lancet Oncol*. 2019;20(6):781-794. doi:10.1016/S1470-2045(19)30152-4

113. Richardson PG, Nagler A, Ben‐Yehuda D, et al. Randomized, placebo-controlled, phase 3 study of perifosine combined with bortezomib and dexamethasone in patients with relapsed, refractory multiple myeloma previously treated with bortezomib. *EJHaem*. 2020;1(1):94-102. doi:10.1002/JHA2.4

114. Rodriguez-Otero P, Ailawadhi S, Arnulf B, et al. Ide-cel or Standard Regimens in Relapsed and Refractory Multiple Myeloma. *N Engl J Med*. 2023;388(11):1002-1014. doi:10.1056/NEJMOA2213614

115. San-Miguel JF, Hungria VTM, Yoon SS, et al. Panobinostat plus bortezomib and dexamethasone versus placebo plus bortezomib and dexamethasone in patients with relapsed or relapsed and refractory multiple myeloma: a multicentre, randomised, double-blind phase 3 trial. *Lancet Oncol*. 2014;15(11):1195-1206. doi:10.1016/S1470-2045(14)70440-1

116. San-Miguel J, Dhakal B, Yong K, et al. Cilta-cel or Standard Care in Lenalidomide-Refractory Multiple Myeloma. *N Engl J Med*. 2023;389(4):335-347. doi:10.1056/NEJMOA2303379

117. Schjesvold FH, Dimopoulos MA, Delimpasi S, et al. Melflufen or pomalidomide plus dexamethasone for patients with multiple myeloma refractory to lenalidomide (OCEAN): a randomised, head-to-head, open-label, phase 3 study. *Lancet Haematol*. 2022;9(2):e98-e110. doi:10.1016/S2352-3026(21)00381-1

118. Sonneveld P, Suciu S, Weijermans P, et al. Cyclosporin A combined with vincristine, doxorubicin and dexamethasone (VAD) compared with VAD alone in patients with advanced refractory multiple myeloma: an EORTC-HOVON randomized phase III study (06914). *Br J Haematol*. 2001;115(4):895-902. doi:10.1046/J.1365-2141.2001.03171.X

119. Spicka I, Ocio EM, Oakervee HE, et al. Randomized phase III study (ADMYRE) of plitidepsin in combination with dexamethasone vs. dexamethasone alone in patients with relapsed/refractory multiple myeloma. *Ann Hematol*. 2019;98(9):2139-2150. doi:10.1007/S00277-019-03739-2

120. Stewart AK, Rajkumar SV, Dimopoulos MA, et al. Carfilzomib, Lenalidomide, and Dexamethasone for Relapsed Multiple Myeloma. *New England Journal of Medicine*. 2015;372(2):142-152. doi:10.1056/NEJMOA1411321/SUPPL_FILE/NEJMOA1411321_DISCLOSURES.PDF

121. Weber DM, Chen C, Niesvizky R, et al. Lenalidomide plus dexamethasone for relapsed multiple myeloma in North America. *N Engl J Med*. 2007;357(21):2133-2142. doi:10.1056/NEJMOA070596

122. Xia Z, Leng Y, Fang B, et al. Aponermin or placebo in combination with thalidomide and dexamethasone in the treatment of relapsed or refractory multiple myeloma (CPT-MM301): a randomised, double-blinded, placebo-controlled, phase 3 trial. *BMC Cancer*. 2023;23(1):1-14. doi:10.1186/S12885-023-11489-8/TABLES/3

123. An Investigational Immuno-therapy Study of Nivolumab, Pomalidomide and Dexamethasone Combinations in Patients With Multiple Myeloma (CheckMate 602). https://clinicaltrials.gov/study/NCT02726581.

Table S1. Studies assessing anti-myeloma treatments in newly diagnosed multiple myeloma

| **Author, year of publication, NCT/EudraCT identifier** | **Study initiation year** | **Follow-up in months** | **Number of patients** | **Transplantation eligibility** | **Treatment type (if reported)** | **Comparison** | **Primary outcome** | **Results statistically significant** |
| --- | --- | --- | --- | --- | --- | --- | --- | --- |
| Horvath, 2019, 01539083 ^1^ | 2012 | 23.2 | 203 | Eligible | Consolidation | Thal,Pre vs Bor,Thal,Pre (after ASCT) | Response | No |
| Kyle, 2009 ^2^ | 1994 | 88.1 | 236 | NS | Consolidation | Mel,Cyclo,Pre,Vcr,carmustine vs Mel,Cyclo,Pre,Vcr,carmustine,High-dose Cyclo,INF | Response, PFS, OS | No |
| Mellqvist, 2013, 00417911 ^3^ | 2005 | 38 | 370 | Eligible | Consolidation | Bor vs Observation (after ASCT) | PFS | No |
| Sonneveld, 2021, 01208766 ^4^ | 2011 | 74.8 | 878 | Eligible | Consolidation | Dex,Bor,Len vs Observation | PFS | Yes |
| Spencer, 2009 ^5^ | 2002 | 64.8 | 243 | Eligible | Consolidation | Thal + Pre(maintenance) vs Pre(maintenance) | PFS, OS | Yes |
| Jacobus, 2016 ^6^ | 2008 | 72 | 48 | All | Consolidation | Dex,Bor,Len vs Dex,Bor | PFS | No |
| Cavo, 2010, 01134484 ^7^ | 2006 | 120 | 480 | Eligible | Induction | Dex,Bor,Thal (+Double Auto+Dex,Bor,Thal consolidation) vs Dex,Thal (+Double Auto+Dex,Thal consolidation) | Response | Yes |
| Cook, 2004 ^8^ | 1996 | 19 | 106 | NS | Induction | Dex,Idarubicin vs Dex,Vcr,Adriamycin | Response | No |
| Dimopoulos, 2003 ^9^ | 1999 | NR | 272 | All | Induction | Dex,Vcr,Doxorubicin vs Dex,Vcr,Liposomal Doxorubicin | Response, Adverse events | No |
| Goldschmidt, 2021, 02495922 ^10^ | 2015 | 49.8 | 564 | Eligible | Induction | Dex,Bor,Len,HDM,ASCT (+ Dex,Bor,Len consolidation + Len maintenance) vs Dex,Bor,Len,HDM,ASCT (+ Dex,Bor,Len,Elotuzumab consolidation + Len,Elotumumab maintenance) vs Dex,Bor,Len,Elotuzumab HDM,ASCT (+ Dex,Bor,Len consolidation + Len maintenance) vs Dex,Bor,Len,Elotuzumab HDM,ASCT (+ Dex,Bor,Len,Elotuzumab consolidation + Len,Elotuzumab maintenance) | PFS | No |
| Goldschmidt, 2022, 03617731 ^11^ | 2018 | 4.2 | 660 | Eligible | Induction | Dex,Bor,Len,Isatuximab vs Dex,Bor,Len | MRD | Yes |
| Harousseau, 2010, 00200681 ^12^ | 2005 | 32.2 | 482 | Eligible | Induction | Dex,Vcr,Doxorubicin vs Dex,Bor vs Dex,Vcr,Doxorubicin +DCEP consolidation vs Dex,Bor + DCEP consolidation | Response | Yes |
| Hungria, 2016, 01532856 ^13^ | 2006 | 37.5 | 82 | Ineligible | Induction | Thal,Mel,Pre vs Dex,Thal,Cyclo vs Dex,Thal | Response | No |
| Jackson, 2021, 49407852 ^14^ | 2013 | 34.5 | 1056 | Eligible | Induction | Dex,Carflizomib,Len,Cyclo vs Dex,Len,Cyclo vs Dex,Thal,Cyclo | PFS, OS | Yes |
| Jackson, 2021, 49407852 ^15^ | 2010 | 50 | 1852 | Ineligible | Induction | Dex,Len,Cyclo vs Dex,Thal,Cyclo | PFS, OS | No |
| Knop, 2017, 01685814 ^16^ | 2012 | NR | 476 | Eligible | Induction | Dex,Len,Adriamycin vs Dex,Bor,Len | Response | No |
| Kumar, 2015 ^17^ | 2009 | 70 | 200 | All | Induction | Len,Low-dose Dex vs Thal,Low-dose Dex | Response, PFS, Adverse events, Quality of life | No |
| Kumar, 2020, 01863550 ^18^ | 2013 | 26 | 1087 | Ineligible | Induction | Dex,Carflizomib,Len vs Dex,Bor,Len | PFS, OS | No |
| Lokhorst, 2010, 06413384 ^19^ | 2001 | 129 | 556 | Eligible | Induction | Dex,Adriamycin,Vcr (+INF maintenance) vs Dex,Thal,Adriamycin (+Thal maintenance) | EFS | Yes |
| Ludwig, 2009, 00205751 ^20^ | 2001 | 28.1 | 289 | Ineligible | Induction | Dex,Thal vs Mel,Pre | PFS, Adverse events | No |
| Ludwig, 2009 (part), 00205751 ^21^ | 2001 | 35 | 128 | Ineligible | Maintenance | Thal,INF vs INF | PFS | Yes |
| Magarotto, 2016, 01093196 ^22^ | 2009 | 39 | 654 | Ineligible | Induction | Len,Mel,Pre vs Len,Cyclo,Pre vs Len,Low-dose Dex | PFS | No |
| Mai, 2015 ^23^  (1 randomization 4 arms – look maintenance) | 2010 | 60.1 | 504 | Eligible | Induction | Dex,Bor,Cyclo vs Dex,Bor,Doxorubicin | Response | Yes |
| Mateos, 2010, 00443235 ^24^ | 2006 | 72 | 260 | Ineligible | Induction | Bor,Mel,Pre vs Bor,Thal,Pre | Response | No |
| Mateos, 2018, 02195479 ^25^ | 2015 | 40.1 | 706 | Ineligible | Induction | Bor,Daratumumab,Mel,Pre vs Bor,Mel,Pre | PFS | Yes |
| Mookerjee, 2017 ^26^ | 2014 | 17.1 | 144 | NS | Induction | Bor,Len,Low-dose Dex vs Len,Low-dose Dex | PFS | No |
| Moreau, 2011, 00910897 ^27^ | 2008 | 32 | 199 | Eligible | Induction | Dex,Bor1.3 mg/m2 vs Dex,Thal,Bor1.0 mg/m2 | Response | No |
| Moreau, 2016 ^28^ | 2013 | 3 | 340 | Eligible | Induction | Dex,Bor,Thal vs Dex,Bor,Cyclo | Response | Yes |
| Moreau, 2019, 02541383 ^29^ | 2015 | 18.8 | 1085 | Eligible | Induction | Dex,Bor,Thal,Daratumumab vs Dex,Bor,Thal | Response | Yes |
| Morgan, 2011 (non-intensive), 68454111 ^30^ | 2003 | 44 | 849 | Ineligible | Induction | Mel,Pre vs Dex,Thal,Cyclo | Response, PFS, OS | Yes |
| Morgan, 2012 (intensive), 68454111 ^31^ | 2003 | 47 | 1111 | Eligible | Induction | Dex,Thal,Cyclo vs Dex,Cyclo,Doxorubicin,Vcr | Response, PFS, OS | No |
| Niesvizky, 2015, 00507416 ^32^ | 2007 | 42.7 | 502 | Ineligible | Induction | Dex,Bor vs Dex,Bor,Thal vs Bor,Mel,Pre | PFS | No |
| Palumbo, 2010, 01063179 ^33^ | 2006 | 54 | 511 | Ineligible | Induction | Bor,Thal,Mel,Pre (+Bor,Thal maintenance) vs Bor,Mel,Pre (no maintenance) | PFS | Yes |
| Ponisch, 2006 ^34^ | 1999 | 48 | 136 | Ineligible | Induction | Bendamustine,Pre vs Mel,Pre | Time to treatment failure | Yes |
| Rajkumar, 2006 ^35^ | 2002 | 4 | 207 | NS | Induction | Dex,Thal vs Dex | Response | Yes |
| Rajkumar, 2008, 00057564 ^36^ | 2003 | 18 | 470 | All | Induction | Dex,Thal vs Dex,Placebo | TTP | Yes |
| Rifkin, 2006 ^37^ | 2001 | 21 | 192 | All | Induction | Low-dose Dex,Vcr,Liposomal Doxorubicin vs Low-dose Dex,Doxorubicin,Vcr | Adverse events, response | Yes |
| Rosinol, 2012, 00461747 ^38^ | 2006 | 70.6 | 390 | Eligible | Induction | Dex,Bor,Thal vs Dex,Thal vs Mel,Cyclo,Pre,Vcr,BCNU/ Dex,Vcr,BCNU,Doxorubicin/Bor | Response | Yes |
| Shustik, 2006 ^39^ | 1995 | 62.4 | 466 | NS | Induction | Mel,Pre vs Dex,Mel | OS | No |
| Straka, 2016, 02288741 ^40^ | 2001 | 624 | 434 | Eligible | Induction | Mel,Double auto,Anthracycline-based induction chemotherapy vs Mel,Double auto | PFS | No |
| Usmani, 2019, 02579863 ^41^ | 2016 | 6.6 | 301 | Ineligible | Induction | Dex,Len,Pembrolizumab vs Dex,Len | PFS | No |
| Zervas, 2007 ^42^ | 2002 | 24 | 232 | NS | Induction | Dex,Vcr,Liposomal Doxorubicin vs Dex,Thal,Vcr,Liposomal Doxorubicin | Response | Yes |
| Jackson, 2019 (part), 49407852 ^43^ | 2010 | 29.7 | 583 | All | Intensification | Dex,Bor,Cyclo vs Observation | PFS, OS | No |
| Attal, 2012, 00430365 ^44^ | 2006 | 60.6 | 614 | Eligible | Maintenance | Len vs Placebo | PFS | Yes |
| Barlogie, 2006 ^45^ | 1993 | 76 | 242 | Eligible | Maintenance | INF vs Observation | OS, response | No |
| Bringhen, 2017 (part), 01093196 ^46^ | 2009 | 63.7 | 402 | Ineligible | Maintenance | Len vs Len,Pre | PFS | No |
| Dimopoulos, 2018, 02181413 ^47^ | 2014 | 31 | 656 | Eligible | Maintenance | Ixazomib vs Placebo | PFS | Yes |
| Dimopoulos, 2020, 02312258 ^48^ | 2015 | 21.1 | 706 | Ineligible | Maintenance | Ixazomib vs Placebo | PFS | Yes |
| Dytfeld, 2023, 02659293 ^49^ | 2016 | 33.8 | 180 | Eligible | Maintenance | Dex,Carflizomib,Len vs Len | PFS | Yes |
| Gay, 2015, 01091831 ^50^ | 2009 | 54.5 | 223 | Eligible | Maintenance | Len,Pre vs Len | PFS | No |
| Giralt, 2020, 00075829 ^51^ | 2003 | 146 | 484 | Eligible | Maintenance | Thal,Dex vs Observation (ASCT assigned patients) | PFS | No |
| Jackson, 2019 (part), 49407852 ^52^ | 2011 | 31 | 1917 | All | Maintenance | Len vs Observation vs Len,Vorinostat | PFS, OS | Yes |
| Mateos, 2012 (part), 00443235 ^53^ | 2006 | 38 | 178 | Ineligible | Maintenance | Bor,Thal vs Bor,Pre | Response | No |
| Moreau, 2021, 02541383 ^54^ | 2016 | 35.4 | 886 | Eligible | Maintenance | Daratumumab vs Observation | PFS | Yes |
| Morgan, 2012 (part), 68454111 ^55^ | 2003 | 72 | 820 | All | Maintenance | Thal vs Observation | PFS, OS | Yes |
| Palumbo, 2014, 00551928 ^56^ | 2007 | 51.2 | 251 | Eligible | Maintenance | Len vs Observation | PFS | Yes |
| Rosinol, 2017, 00461747 ^57^ | 2006 | 58.6 | 271 | Eligible | Maintenance | Bor,Thal vs Thal vs Alfa2-IFN | PFS | Yes |
| Schaar, 2005 ^58^ | 1991 | 97 | 90 | NS | Maintenance | INF vs Observation | PFS, OS | No |
| Shustik, 2004 (part) ^59^ | 1995 | 61.2 | 307 | NS | Maintenance | Dex vs Observation | OS | No |
| Barlogie, 2006, 00083551 ^60^ | 1998 | 87 | 668 | Eligible | Not specified | Thal(Induction + Double auto + consolidation + maintenance) vs no Thal(Induction + Double auto + consolidation + maintenance) | EFS | Yes |
| Beksac, 2011, 00934154 ^61^ | 2006 | 23 | 122 | Ineligible | Not specified | Thal,Mel,Pre vs Mel,Pre | Response, Adverse events | Yes |
| Benboubker, 2014, 00689936 ^62^ | 2008 | 67 | 1623 | Ineligible | Not specified | Thal,Mel,Pre vs Dex,Len (fix dose) vs Dex,Len (continuous) | PFS | Yes |
| CL, 00097981 ^63^ | 2005 | 24 | 225 | NS | Not specified | Dex,Thal vs Dex,Thal,DOXIL | Response | No |
| Dimopoulos, 2022, 01335399 ^64^ | 2011 | 70.6 | 748 | Ineligible | Not specified | Dex,Len,Elotuzumab vs Dex,Len | PFS | No |
| Durie, 2017, 00644228 ^65^ | 2008 | 84 | 525 | NS | Not specified | Dex,Bor,Len vs Dex,Len | PFS | Yes |
| Facon, 2019, 01818752 ^66^ | 2013 | 22 | 955 | Ineligible | Not specified | Carflizomib,Mel,Pre vs Bor,Mel,Pre | PFS | No |
| Facon, 2019, 02252172 ^67^ | 2015 | 64.5 | 737 | Ineligible | Not specified | Dex,Len,Daratumumab vs Dex,Len | PFS | Yes |
| Facon, 2021, 01850524 ^68^ | 2013 | 55.8 | 705 | Ineligible | Not specified | Dex,Ixazomib,Len vs Dex,Len,Placebo | PFS | No |
| Fu, 2023, 03217812 ^69^ | 2017 | 12.3 | 220 | Ineligible | Not specified | Bor,Daratumumab,Mel,Pre vs Bor,Mel,Pre | Response | Yes |
| Hulin, 2009 ^70^ | 2002 | 47.5 | 229 | Ineligible | Not specified | Thal,Mel,Pre vs Mel,Placebo,Pre | OS | Yes |
| Palumbo, 2006, 00232934 ^71^ | 2002 | 38.1 | 331 | Ineligible | Not specified | Thal,Mel,Pre vs Mel,Pre | EFS, response | Yes |
| Palumbo, 2012, 00405756 ^72^ | 2007 | 48 | 459 | Ineligible | Not specified | Len,Mel,Pre, + Len maintenance vs Len,Mel,Pre + Placebo maintenance vs Mel,Pre,Placebo + Placebo maintenance | PFS | Yes |
| San Miguel, 2008, 00111319 ^73^ | 2004 | 60.1 | 682 | Ineligible | Not specified | Mel,Pre vs Bor,Mel,Pre | TTP | Yes |
| Sonneveld, 2012, 2004-000944-26 ^74^ | 2005 | 137 | 827 | Eligible | Not specified | Dex,Vcr,Doxorubicin + ASCT + Thal maintenance vs Dex,Bor,Doxorubicin + ASCT + Bor maintenance | PFS | Yes |
| Stewart, 2015, 00602641 ^75^ | 2008 | 40.7 | 306 | Ineligible | Not specified | Thal,Mel,Pre induction + Thal maintenance vs Len,Mel,Pre induction + Len maintenance | PFS | No |
| Takenaka, 2004 ^76^ | 1993 | 43 | 210 | NS | Not specified | Mel,Cyclo,Pre,Vcr,Ranimustine vs Mel,Cyclo,Pre,Vcr | OS | No |
| Waage, 2010, 00218855 ^77^ | 2002 | 42 | 363 | Ineligible | Not specified | Thal,Mel,Pre vs Mel,Placebo,Pre | OS | No |
| Wijermans, 2010, 90692740 ^78^ | 2002 | 39 | 333 | Ineligible | Not specified | Thal,Mel,Pre vs Mel,Pre | EFS | Yes |
| Zonder, 2010, 00064038 ^79^ | 2004 | 47.2 | 198 | Ineligible | Not specified | Dex,Len vs Dex,Placebo | PFS | Yes |
| Zweegman, 2016, 2007-004007-34 ^80^ | 2009 | 36 | 637 | Ineligible | Not specified | Thal,Mel,Pre induction + Thal maintenance vs Len,Mel,Pre induction + Len maintenance | PFS | No |
| Maiolino, 2012, 01296503 ^81^ | 2003 | 27 | 108 | Eligible | Maintenance | Dex vs Dex,Thal | PFS | Yes |
| Stewart, 2013, 00049673 ^82^ | 2002 | 73.2 | 332 | Eligible | Maintenance | Thal,Pre vs Observation | OS | No |
| Offidani, 2008 ^83^ | NR | 30 | 103 | NS | Maintenance | Dex,Thal vs Dex,INF | TTP | Yes |
| McCarthy, 2012, 00114101 ^84^ | 2005 | 91 | 460 | Eligible | Maintenance | Len vs Placebo | TTP | Yes |

## Table S2. Studies with antimyeloma treatment in Relapsed/Refractory Multiple Myeloma

| **Author, year of publication, NCT/EudraCT** | **Study initiation year** | **Follow-up in months** | **No of patients** | **Lines of previous treatments** | **Transplantation eligibility** | **Treatment type (if reported)** | **Comparison** | **Primary Outcome** | **Results (statistically significant)** |
| --- | --- | --- | --- | --- | --- | --- | --- | --- | --- |
| Montefusco, 2020, 2010-021557-40 ^85^ | 2011 | 34 | 155 | 1 | All | Induction | Dex,Bor,Cyclo vs Dex,Len,Cyclo | Response | No |
| Attal, 2019, 02990338 ^86^ | 2017 | 35.3 | 307 | ≥2 | NS | Not specified | Dex,Pomalidomide,Isatuximab vs Dex,Pomalidomide | PFS | Yes |
| Chanan-Khan, 2009 ^87^ | 2001 | 24 | 224 | ≥ 1 | NS | Not specified | Dex,Oblimersen vs Dex | TTP | No |
| Dimopoulos, 2007, 00424047 ^88^ | 2003 | 16.4 | 351 | ≥ 1 | NS | Not specified | Dex,Len vs Dex,Placebo | TTP | Yes |
| Dimopoulos, 2013, 00773747 ^89^ | 2008 | 14.2 | 637 | 1 - 3 | NS | Not specified | Bor,Vorinostat vs Bor,Placebo | PFS | Yes |
| Dimopoulos, 2016, 01568866 ^90^ | 2012 | 44.3 | 929 | 1 - 3 | NS | Not specified | Dex,Carflizomib vs Dex,Bor | PFS | Yes |
| Dimopoulos, 2016, 02076009 ^91^ | 2014 | 79.7 | 569 | ≥ 1 | NS | Not specified | Dex,Len,Daratumumab vs Dex,Len | PFS | Yes |
| Dimopoulos, 2020, 03158688 ^92^ | 2017 | 50 | 466 | 1 - 3 | NS | Not specified | Dex,Carflizomib,Daratumumab vs Dex,Carflizomib | PFS | Yes |
| Dimopoulos, 2021, 03180736 ^93^ | 2017 | 39.6 | 304 | ≥ 1 | NS | Not specified | Dex,Pomalidomide,Daratumumab vs Dex,Pomalidomide | PFS | Yes |
| Dimopoulos, 2023, 04162210 ^94^ | 2020 | 11.5 | 325 | ≥2 | NS | Not specified | belantamab mafodotin 2.5 mg/kg frozen liquid vs Dex,Pomalidomide | PFS | No |
| Friedenberg, 2006 ^95^ | 1997 | 31.1 | 94 |  | NS | Not specified | Dex,Vcr,Doxorubicin,Valspodar vs Dex,Doxorubicin,Vcr | Response, OS | No |
| Garderet, 2012, 2005-001628-35 ^96^ | 2006 | 30 | 269 | at least one auto | NS | Not specified | Dex,Bor,Thal vs Dex,Thal | TTP | Yes |
| Grosicki, 2020, 03110562 ^97^ | 2017 | 16.5 | 402 | 1 - 3 | NS | Not specified | Dex,Bor,Selinexor vs Dex,Bor | PFS | Yes |
| Hajek, 2017, 01302392 ^98^ | 2010 | 29.8 | 315 | >=3 | NS | Not specified | Carflizomib vs Low-dose corticosteroids,Optional Cyclo | OS | No |
| Hjorth, 2012, 00602511 ^99^ | 2007 | 16 | 131 | ≥ 1 | NS | Not specified | Dex,Thal vs Dex,Bor | PFS | No |
| Kropff, 2017, 00813150 ^100^ | 2008 | 24 | 96 | 1 - 3 | NS | Not specified | Dex,Bor vs Dex,Bor,Cyclo | TTP | No |
| Kumar, 2020, 02755597 ^101^ | 2016 | 45.6 | 291 | 1 - 3 | NS | Not specified | Dex,Bor,Venetoclax vs Dex,Bor,Placebo | PFS | Yes |
| Lonial, 2015, 01239797 ^102^ | 2011 | 70.6 | 646 | 1 - 3 | NS | Not specified | Dex,Len,Elotuzumab vs Dex,Len | Response, PFS | Yes |
| Lu, 2021, 03234972 ^103^ | 2017 | 25.1 | 211 | ≥ 1 | NS | Not specified | Dex,Bor,Daratumumab vs Dex,Bor | PFS | Yes |
| Mateos, 2019, 02576977 ^104^ | 2016 | 8.1 | 249 | >1 | NS | Not specified | Dex,Pomalidomide,Pembrolizumab vs Dex,Pomalidomide | PFS, OS | No |
| Miguel, 2013, 01311687 ^105^ | 2011 | 15.4 | 455 | ≥2 | NS | Not specified | Pomalidomide,Low-dose Dex vs High-dose Dex | PFS | Yes |
| Moreau, 2016, 01564537 ^106^ | 2012 | 85 | 722 | 1 - 3 | NS | Not specified | Dex,Ixazomib,Len vs Dex,Len,Placebo | PFS | Yes |
| Moreau, 2021, 03275285 ^107^ | 2017 | 44 | 302 | 1 - 3 | NS | Not specified | Dex,Carflizomib,Isatuximab vs Dex,Carflizomib | PFS | Yes |
| Orlowski, 2007, 00103506 ^108^ | 2004 | 103 | 646 | ≥ 1 | NS | Not specified | Bor,Pegylated liposomal doxorubicin vs Bor | TTP | Yes |
| Palumbo, 2016, 02136134 ^109^ | 2014 | 72.6 | 498 | ≥ 1 | NS | Not specified | Dex,Bor,Daratumumab vs Dex,Bor | PFS | Yes |
| Pour, 2023, 04649060 ^110^ | 2020 | 7.1 | 54 | >=3 | NS | Not specified | Dex,Daratumumab,Melflufen vs Dex,Daratumumab | PFS | Yes |
| Richardson, 2005, 00048230 ^111^ | 2002 | 22 | 669 | 1 - 3 | NS | Not specified | Bor vs Dex | TTP | Yes |
| Richardson, 2019, 01734928 ^112^ | 2013 | 16.4 | 559 | 1 - 3 | NS | Not specified | Dex,Bor,Pomalidomide vs Dex,Bor | PFS | Yes |
| Richardson, 2020, 01002248 ^113^ | 2010 | 7.75 | 135 | 1 - 4 | NS | Not specified | Dex,Bor,Perifosine vs Dex,Bor,Placebo | PFS | No |
| Rodriguez‑Otero, 2023, 03651128 ^114^ | 2019 | 18.6 | 386 | 2-4 | NS | Not specified | Idecabtagene vicleucel vs 1 of 5 standard regimens chosen at investigators' discresion (Dara,Pom,Dex / Carflizomib,Dex / Elotuzumab,Pom,Dex / Ixazomib,Len,Dex / Dara,Bor,Dex) | PFS | Yes |
| San-Miguel, 2014, 01023308 ^115^ | 2010 | 6.47 | 768 | 1 - 3 | NS | Not specified | Dex,Bor,Panobinostat vs Dex,Bor,Placebo | PFS | Yes |
| San-Miguel, 2023, 04181827 ^116^ | 2020 | 15.9 | 419 | 1 - 3 | NS | Not specified | Ciltacabtagene autoleucel vs Physician’s choice (1 of 3: Pomalidomide,Bor,Dex (PVd) / Daratumumab,Pomalidomide,Dex (DPd) / single cilta-cel infusion, administered after the physician’s choice of bridging therapy (PVd or DPd)) | PFS | Yes |
| Schjesvold, 2022, 03151811 ^117^ | 2017 | 16.3 | 495 | 2-4 | NS | Not specified | Dex,Melflufen vs Dex,Pomalidomide | PFS | Yes |
| Sonneveld, 2001 ^118^ | NR | 49 | 81 | ≥ 1 | NS | Not specified | Dex,Vcr,Doxorubicin,Cyclosporin A vs Dex,Doxorubicin,Vcr | Response | No |
| Spicka, 2019 01102426 ^119^ | 2010 | NR | 255 | 3-6 | NS | Not specified | Dex,Plitidepsin vs Dex | PFS | Yes |
| Stewart, 2015, 01080391 ^120^ | 2010 | 67.1 | 792 | 1 - 3 | NS | Not specified | Dex,Carflizomib,Len vs Dex,Len | PFS | Yes |
| Weber, 2007, 00056160 ^121^ | 2003 | 17.6 | 353 | ≥ 1 | NS | Not specified | Dex,Len vs Dex,Placebo | TTP | Yes |
| Xia, 2023 ^122^ | 2015 | 17.2 | 417 | ≥2 | NS | Not specified | Dex,Thal,Aponermin vs Dex,Thal,Placebo | PFS | Yes |
| CL, 02726581^123^{, 2016 #30941}{, 2016 #30941} | 2016 | 64 | 170 | ≥2 | NS | Not specified | Nivolumad,Pom,Dex vs Pom,Dex vs Nivolumab,Elotuzumab,Pom,Dex | PFS | No |

**Tables’ Abbreviations**: ABSC: Autologous blood stem cell, allo-HCT: allogeneic hematopoietic cell transplantation, ASCT: autologous stem cell transplantation, ATO: Arsenic trioxide, auto: Autologous stem cell transplantation, Bor: Bortezomib, CL: Clinicaltrials.gov, Cyclo: Cyclophosphamide, DCEP: Dexamethasone, cyclophosphamide, etoposide, cisplatin, Dex: Dexamethasone, DoR: Duration of response, EFS: Event free survival, HDM: High dose melphalan, HDT: High-dose therapy, HSCT: Hematopoietic stem cell transplantation, INF: Interferon, Len: Lenalidomide, Μel: Melphalan, M-VTD-PACE: Melphalan-Bortezomib, Thalidomide, Dexamethasone-Cisplatin, Adriamycin, Cyclophosphamide, Etoposide), NA: Not applicable, NR: Not reported, NS: Not specified, OS: Overall survival, PFS: Progression free survival, Pre: Prednisone/Prednisolone, SCT: Stem cell transplantation, Thal: Thalidomide, TRM: Treatment related mortality, TTP: Time to progression, VAD: Vincristine, adriamycin, dexamethasone, Vcr: Vincristine, VRD: Bortezomib, Lenalidomide, Dexamethasone, vs: Versus, VTD: Bortezomib, Thalidomide, Dexamethasone

# Additional Supplementary Results

# Regression modeling

Univariable and multivariable logistic regression was used to assess the association of covariates with the statistical significance of each comparison. The variables for the inclusion in the multivariable model were selected based on substantive interest and not based on univariable pre-screening. For continuous covariates included in the multivariable model, AIC-guided selection was used to select the best-fitting transformation. Missingness in study characteristics was treated as a separate category for categorical characteristics, while for continuous predictors (age), the mean value for non-missing observations was imputed and a missingness indicator was included in the model.


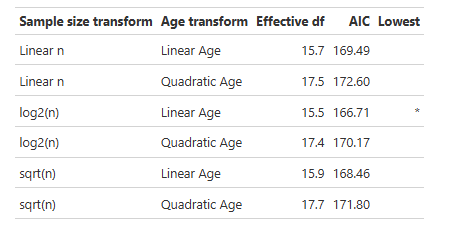


## Variance inflation

Multi-collinearity assessment using the variance inflation factor (VIF), using generalized VIFs for multiple degree-of-freedom predictors, not suggesting concern (all values < 2).


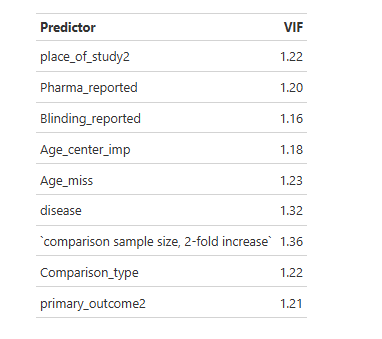


## Sensitivity analyses without influential observations

The effect of potential outliers was examined both by removing the 5 comparisons with the highest Cook’s distance (actual impact on coefficients) and the 5 comparisons with the highest leverage (potential impact due to covariates).


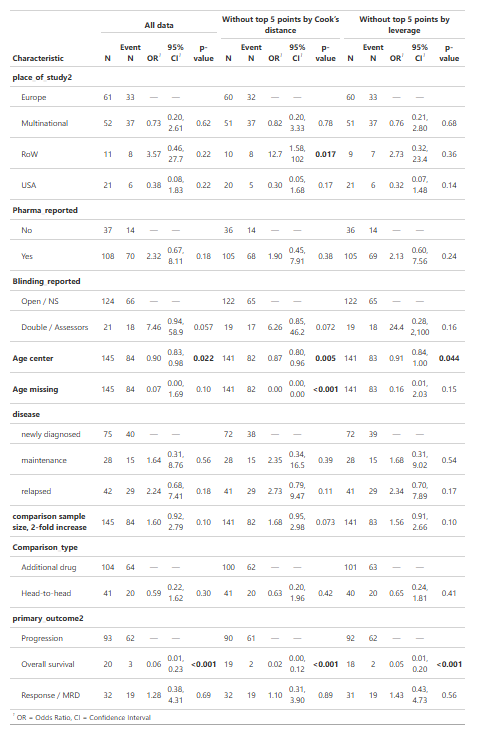


## Visualization of model fit

## Effect plots for variables included in the model


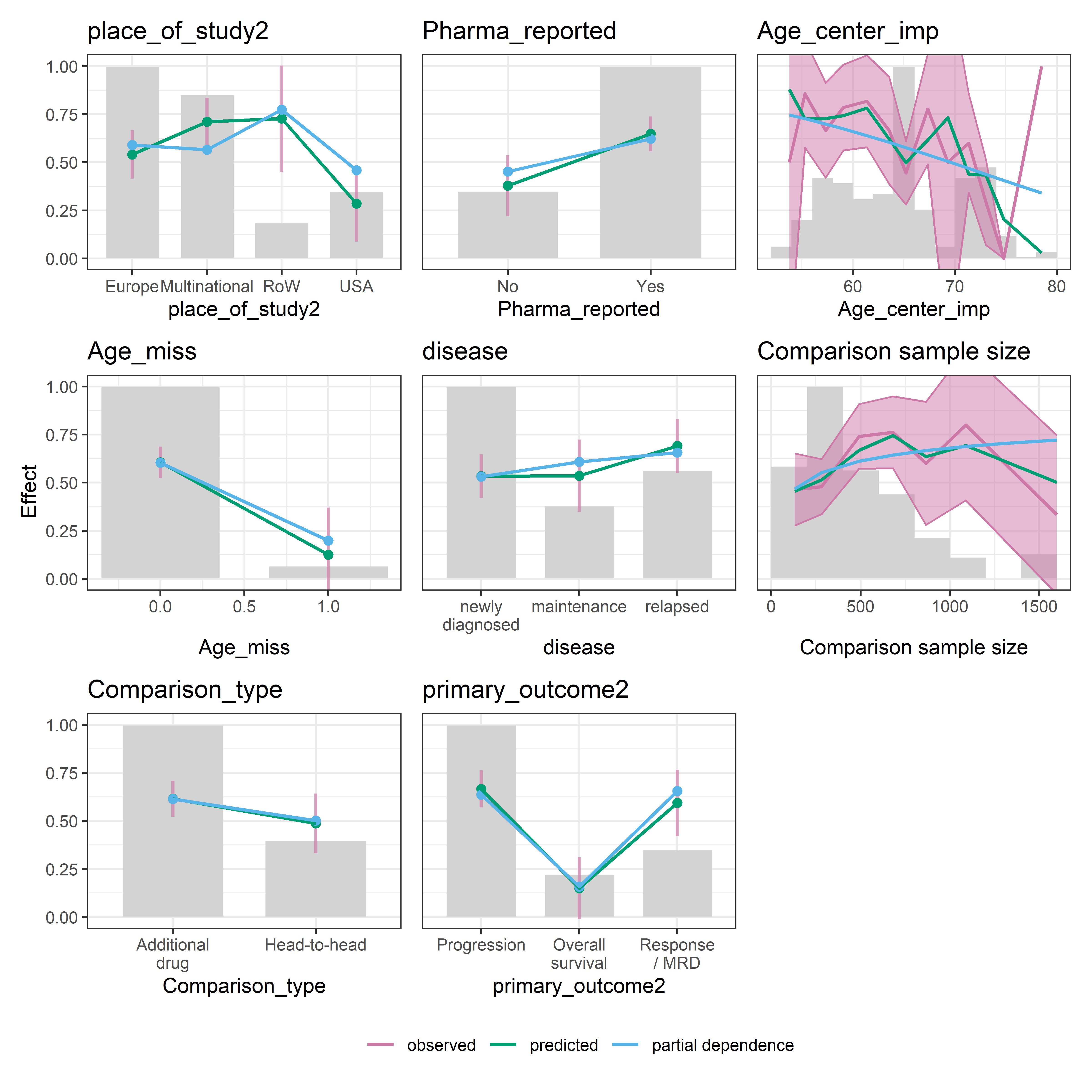


## Goodness-of-fit plots for variables not included in the model


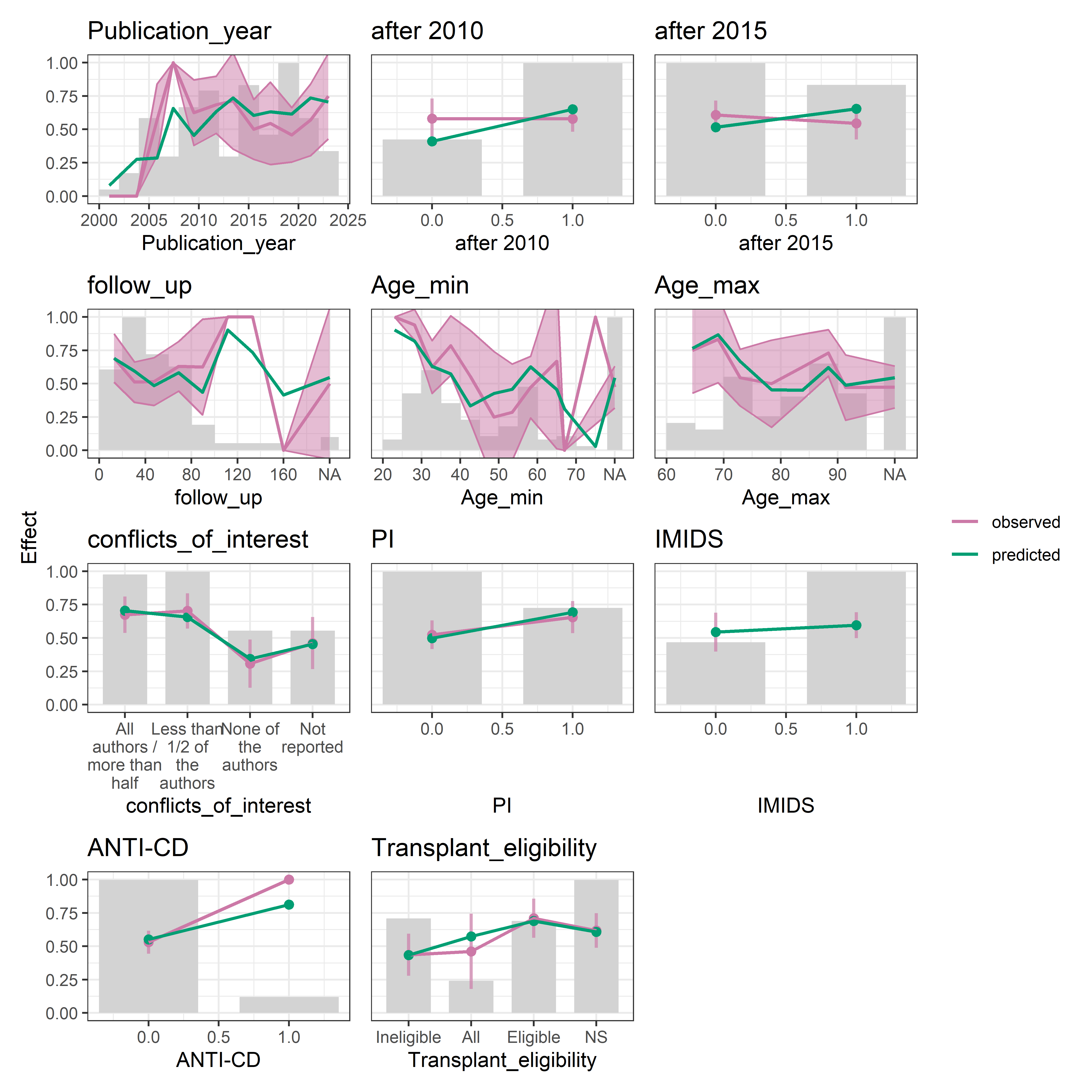


## Explore effect of publication year


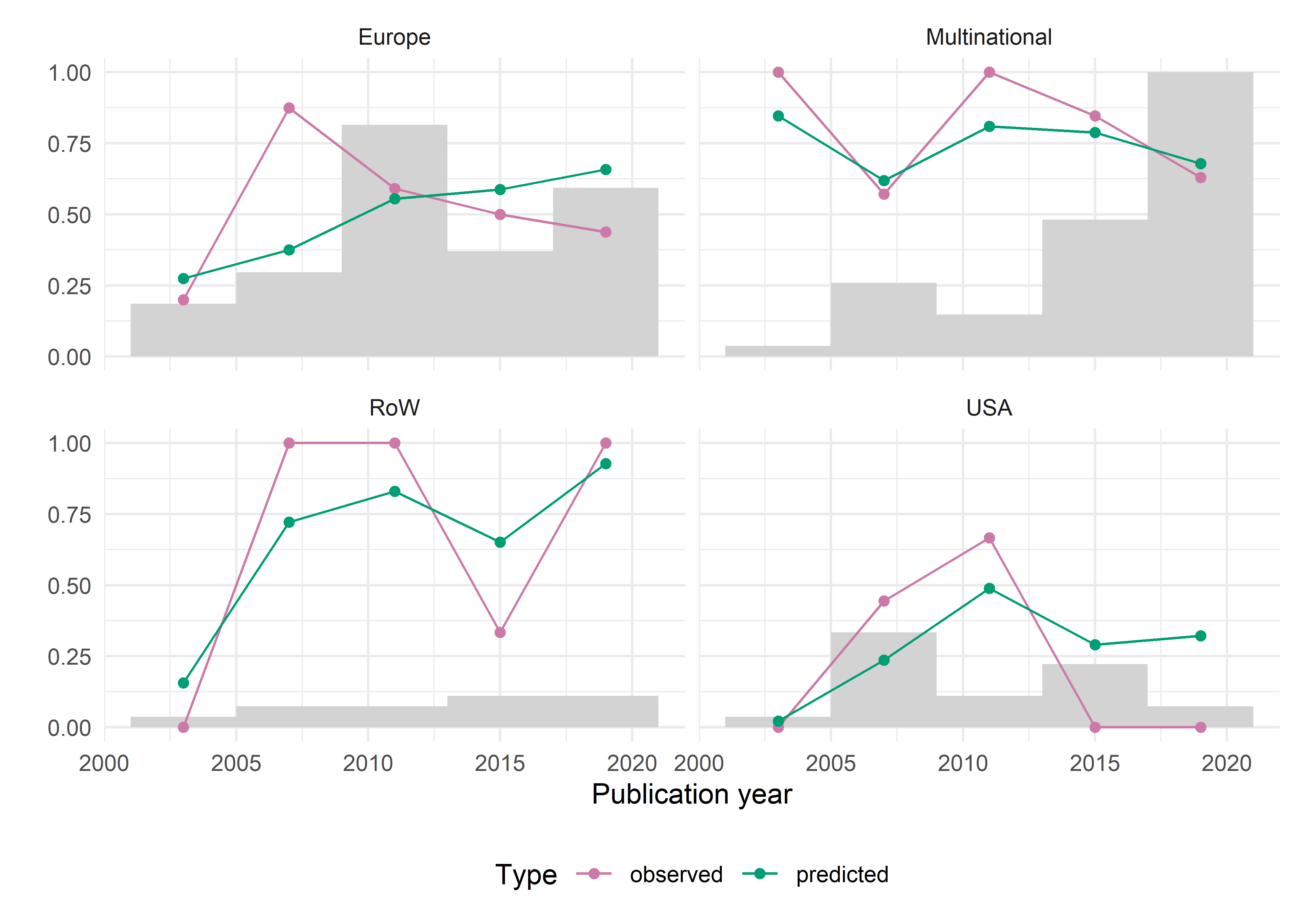


# Subgroup analyses

## Subgroup analysis by disease setting

### Univariate predictors of positive comparisons

|  | **newly diagnosed/maintenance** | | | **relapsed** | | |
| --- | --- | --- | --- | --- | --- | --- |
| **Characteristic** | **0** N = 48*^1^* | **1** N = 55*^1^* | **p-value** | **0** N = 13*^1^* | **1** N = 29*^1^* | **p-value** |
| **Publication_year** | 2,015.0 (2,010.0, 2,019.0) | 2,012.0 (2,009.0, 2,017.0) | 0.30*^2^* | 2,017.0 (2,009.0, 2,020.0) | 2,016.0 (2,014.0, 2,021.0) | 0.60*^2^* |
| **after 2010** |  |  | 0.37*^3^* |  |  | 0.25*^3^* |
| 0 | 14 (40%) | 21 (60%) |  | 4 (50%) | 4 (50%) |  |
| 1 | 34 (50%) | 34 (50%) |  | 9 (26%) | 25 (74%) |  |
| **after 2015** |  |  | 0.21*^3^* |  |  | 0.98*^3^* |
| 0 | 26 (41%) | 37 (59%) |  | 5 (31%) | 11 (69%) |  |
| 1 | 22 (55%) | 18 (45%) |  | 8 (31%) | 18 (69%) |  |
| **follow_up** | 43 (31, 71) | 47 (31, 65) | 0.80*^2^* | 24 (8, 31) | 25 (16, 50) | 0.15*^2^* |
| Unknown | 1 | 1 |  | 1 | 1 |  |
| **place_of_study2** |  |  | 0.16*^3^* |  |  | **0.043***^3^* |
| Europe | 24 (43%) | 32 (57%) |  | 4 (80%) | 1 (20%) |  |
| Multinational | 8 (40%) | 12 (60%) |  | 7 (22%) | 25 (78%) |  |
| RoW | 3 (33%) | 6 (67%) |  | 0 (0%) | 2 (100%) |  |
| USA | 13 (72%) | 5 (28%) |  | 2 (67%) | 1 (33%) |  |
| **Pharma_reported** |  |  | 0.32*^3^* |  |  | **<0.001***^3^* |
| No | 17 (55%) | 14 (45%) |  | 6 (100%) | 0 (0%) |  |
| Yes | 31 (43%) | 41 (57%) |  | 7 (19%) | 29 (81%) |  |
| **Blinding_reported** |  |  | 0.052*^3^* |  |  | 0.11*^3^* |
| Open / NS | 46 (50%) | 46 (50%) |  | 12 (38%) | 20 (63%) |  |
| Double / Assessors | 2 (18%) | 9 (82%) |  | 1 (10%) | 9 (90%) |  |
| **sample size** | 370 (236, 654) | 470 (232, 706) | 0.79*^2^* | 155 (96, 249) | 455 (307, 646) | **<0.001***^2^* |
| **Age_center** | 66.0 (60.5, 73.0) | 60.0 (57.5, 71.0) | **0.004***^2^* | 65.00 (65.00, 68.00) | 64.30 (62.00, 66.00) | **0.044***^2^* |
| Unknown | 4 | 1 |  | 3 | 0 |  |
| **Comparison_type** |  |  | 0.37*^3^* |  |  | 0.35*^3^* |
| Additional drug | 31 (44%) | 40 (56%) |  | 9 (27%) | 24 (73%) |  |
| Head-to-head | 17 (53%) | 15 (47%) |  | 4 (44%) | 5 (56%) |  |
| **Comparison sample size** | 335 (236, 637) | 470 (220, 706) | 0.78*^2^* | 155 (96, 249) | 455 (307, 646) | **<0.001***^2^* |
| **primary_outcome2** |  |  | **0.007***^3^* |  |  | **0.001***^3^* |
| Progression | 24 (41%) | 34 (59%) |  | 7 (20%) | 28 (80%) |  |
| Overall survival | 14 (82%) | 3 (18%) |  | 3 (100%) | 0 (0%) |  |
| Response / MRD | 10 (36%) | 18 (64%) |  | 3 (75%) | 1 (25%) |  |
| **# of significance tests** | 1 (1, 2) | 1 (1, 2) | 0.18*^2^* | 1 (1, 2) | 1 (1, 1) | 0.19*^2^* |
| **Transplant_eligibility** |  |  | 0.054*^3^* |  |  |  |
| Ineligible | 22 (56%) | 17 (44%) |  | 0 (NA%) | 0 (NA%) |  |
| All | 6 (50%) | 6 (50%) |  | 1 (100%) | 0 (0%) |  |
| Eligible | 11 (29%) | 27 (71%) |  | 0 (NA%) | 0 (NA%) |  |
| NS | 9 (64%) | 5 (36%) |  | 12 (29%) | 29 (71%) |  |
| **PI** |  |  | 0.44*^3^* |  |  | 0.27*^3^* |
| 0 | 32 (50%) | 32 (50%) |  | 8 (40%) | 12 (60%) |  |
| 1 | 16 (41%) | 23 (59%) |  | 5 (23%) | 17 (77%) |  |
| **IMIDS** |  |  | 0.41*^3^* |  |  | 0.63*^3^* |
| 0 | 14 (54%) | 12 (46%) |  | 7 (35%) | 13 (65%) |  |
| 1 | 34 (44%) | 43 (56%) |  | 6 (27%) | 16 (73%) |  |
| **ANTI-CD** |  |  | 0.065*^3^* |  |  | **0.020***^3^* |
| 0 | 48 (49%) | 50 (51%) |  | 13 (41%) | 19 (59%) |  |
| 1 | 0 (0%) | 5 (100%) |  | 0 (0%) | 10 (100%) |  |
| *^1^* Median (Q1, Q3); n (%) | | | | | | |
| *^2^* Design-based KruskalWallis test | | | | | | |
| *^3^* Pearson’s X^2: Rao & Scott adjustment | | | | | | |
|  | | | | | | |

## Subgroup analysis by outcome type

### Univariate predictors of positive comparisons

| **Characteristic** | **Response / MRD** | | | **TTE outcome** | | |
| --- | --- | --- | --- | --- | --- | --- |
|  | **0** N = 13*^1^* | **1** N = 19*^1^* | **p-value** | **0** N = 48*^1^* | **1** N = 65*^1^* | **p-value** |
| **Publication_year** | 2,010.0 (2,006.0, 2,016.0) | 2,012.0 (2,010.0, 2,016.0) | 0.24*^2^* | 2,016.0 (2,011.0, 2,019.0) | 2,015.0 (2,010.0, 2,019.0) | 0.56*^2^* |
| **after 2010** |  |  | 0.10*^3^* |  |  | 0.31*^3^* |
| 0 | 8 (57%) | 6 (43%) |  | 10 (34%) | 19 (66%) |  |
| 1 | 5 (28%) | 13 (72%) |  | 38 (45%) | 46 (55%) |  |
| **after 2015** |  |  | 0.84*^3^* |  |  | 0.41*^3^* |
| 0 | 10 (42%) | 14 (58%) |  | 21 (38%) | 34 (62%) |  |
| 1 | 3 (38%) | 5 (63%) |  | 27 (47%) | 31 (53%) |  |
| **follow_up** | 34 (24, 49) | 32 (19, 70) | 0.45*^2^* | 43 (28, 61) | 44 (25, 65) | 0.72*^2^* |
| Unknown | 1 | 1 |  | 1 | 1 |  |
| **place_of_study2** |  |  | 0.54*^3^* |  |  | **0.006***^3^* |
| Europe | 7 (35%) | 13 (65%) |  | 21 (51%) | 20 (49%) |  |
| Multinational | 2 (67%) | 1 (33%) |  | 13 (27%) | 36 (73%) |  |
| RoW | 1 (25%) | 3 (75%) |  | 2 (29%) | 5 (71%) |  |
| USA | 3 (60%) | 2 (40%) |  | 12 (75%) | 4 (25%) |  |
| **Pharma_reported** |  |  | 0.15*^3^* |  |  | **0.027***^3^* |
| No | 7 (58%) | 5 (42%) |  | 16 (64%) | 9 (36%) |  |
| Yes | 6 (30%) | 14 (70%) |  | 32 (36%) | 56 (64%) |  |
| **Blinding_reported** |  |  |  |  |  | **0.005***^3^* |
| Open / NS | 13 (41%) | 19 (59%) |  | 45 (49%) | 47 (51%) |  |
| Double / Assessors | 0 (NA%) | 0 (NA%) |  | 3 (14%) | 18 (86%) |  |
| **sample size** | 199 (106, 236) | 386 (220, 646) | **<0.001***^2^* | 370 (236, 654) | 466 (271, 669) | 0.57*^2^* |
| **Age_center** | 64.0 (58.0, 71.5) | 59.0 (57.1, 66.0) | 0.23*^2^* | 67.6 (64.6, 73.0) | 64.0 (59.0, 67.0) | **0.003***^2^* |
| Unknown | 2 | 0 |  | 5 | 1 |  |
| **relapsed** |  |  | 0.15*^3^* |  |  | **0.022***^3^* |
| newly diagnosed/maintenance | 10 (36%) | 18 (64%) |  | 38 (51%) | 37 (49%) |  |
| relapsed | 3 (75%) | 1 (25%) |  | 10 (26%) | 28 (74%) |  |
| **Comparison_type** |  |  | 0.95*^3^* |  |  | 0.066*^3^* |
| Additional drug | 7 (41%) | 10 (59%) |  | 33 (38%) | 54 (62%) |  |
| Head-to-head | 6 (40%) | 9 (60%) |  | 15 (58%) | 11 (42%) |  |
| **Comparison sample size** | 199 (106, 236) | 340 (220, 646) | **<0.001***^2^* | 335 (236, 637) | 466 (269, 669) | 0.40*^2^* |
| **# of significance tests** | 1 (1, 1) | 1 (1, 2) | 0.59*^2^* | 1 (1, 2) | 1 (1, 1) | **0.012***^2^* |
| **Transplant_eligibility** |  |  | 0.38*^3^* |  |  | 0.075*^3^* |
| Ineligible | 3 (43%) | 4 (57%) |  | 19 (59%) | 13 (41%) |  |
| All | 2 (50%) | 2 (50%) |  | 5 (56%) | 4 (44%) |  |
| Eligible | 3 (23%) | 10 (77%) |  | 8 (32%) | 17 (68%) |  |
| NS | 5 (63%) | 3 (38%) |  | 16 (34%) | 31 (66%) |  |
| **PI** |  |  | 0.49*^3^* |  |  | 0.22*^3^* |
| 0 | 8 (47%) | 9 (53%) |  | 32 (48%) | 35 (52%) |  |
| 1 | 5 (33%) | 10 (67%) |  | 16 (35%) | 30 (65%) |  |
| **IMIDS** |  |  | 0.16*^3^* |  |  | 0.91*^3^* |
| 0 | 6 (60%) | 4 (40%) |  | 15 (42%) | 21 (58%) |  |
| 1 | 7 (32%) | 15 (68%) |  | 33 (43%) | 44 (57%) |  |
| **ANTI-CD** |  |  | 0.24*^3^* |  |  | **<0.001***^3^* |
| 0 | 13 (43%) | 17 (57%) |  | 48 (48%) | 52 (52%) |  |
| 1 | 0 (0%) | 2 (100%) |  | 0 (0%) | 13 (100%) |  |
| **primary_outcome3** |  |  |  |  |  | **<0.001***^3^* |
| Response / MRD | 13 (41%) | 19 (59%) |  |  |  |  |
| Overall survival |  |  |  | 17 (85%) | 3 (15%) |  |
| Progression |  |  |  | 31 (33%) | 62 (67%) |  |
| *^1^* Median (Q1, Q3); n (%) | | | | | | |
| *^2^* Design-based KruskalWallis test | | | | | | |
| *^3^* Pearson’s X^2: Rao & Scott adjustment | | | | | | |

| **Response/MRD** | | | | | | | | | | |
| --- | --- | --- | --- | --- | --- | --- | --- | --- | --- | --- |
| **Characteristic** | **Univariable models** | | | | | **Multivariable model** | | | | |
|  | **N** | **Event N** | **OR** | **95% CI** | **p-value** | **N** | **Event N** | **OR** | **95% CI** | **p-value** |
| **Publication_year** | 32 | 19 | 1.09 | 0.95, 1.26 | 0.21 |  |  |  |  |  |
| **after 2010** | 32 | 19 | 3.47 | 0.76, 15.8 | 0.10 |  |  |  |  |  |
| **after 2015** | 32 | 19 | 1.19 | 0.20, 7.05 | 0.84 |  |  |  |  |  |
| **place_of_study2** |  |  |  |  |  |  |  |  |  |  |
| Europe | 20 | 13 | — | — |  | 20 | 13 | — | — |  |
| Multinational | 3 | 1 | 0.27 | 0.02, 4.27 | 0.34 | 3 | 1 | 0.01 | 0.00, 0.39 | **0.018** |
| RoW | 4 | 3 | 1.62 | 0.12, 22.4 | 0.71 | 4 | 3 | 16,621 | 15.2, 18,208,712 | **0.009** |
| USA | 5 | 2 | 0.36 | 0.04, 3.20 | 0.34 | 5 | 2 | 7.43 | 0.08, 714 | 0.37 |
| **Pharma_reported** |  |  |  |  |  |  |  |  |  |  |
| No | 12 | 5 | — | — |  | 12 | 5 | — | — |  |
| Yes | 20 | 14 | 3.27 | 0.61, 17.4 | 0.16 | 20 | 14 | 1.09 | 0.06, 20.9 | 0.95 |
| **relapsed** |  |  |  |  |  |  |  |  |  |  |
| newly diagnosed/maintenance | 28 | 18 | — | — |  | 28 | 18 | — | — |  |
| relapsed | 4 | 1 | 0.19 | 0.01, 2.35 | 0.18 | 4 | 1 | 1.41 | 0.18, 11.2 | 0.73 |
| **Age_min** | 18 | 12 | 0.95 | 0.87, 1.03 | 0.20 |  |  |  |  |  |
| **Age_max** | 18 | 12 | 0.99 | 0.88, 1.12 | 0.92 |  |  |  |  |  |
| **Age center** | 32 | 19 | 0.92 | 0.80, 1.05 | 0.20 | 32 | 19 | 0.84 | 0.69, 1.02 | 0.069 |
| **Comparison sample size, 2-fold increase** | 32 | 19 | 9.53 | 2.19, 41.5 | **0.004** | 32 | 19 | 599 | 6.22, 57,737 | **0.009** |
| **Comparison_type** |  |  |  |  |  |  |  |  |  |  |
| Additional drug | 17 | 10 | — | — |  | 17 | 10 | — | — |  |
| Head-to-head | 15 | 9 | 1.05 | 0.22, 5.04 | 0.95 | 15 | 9 | 0.41 | 0.02, 9.02 | 0.55 |
| **follow_up** | 30 | 18 | 1.00 | 0.97, 1.02 | 0.77 |  |  |  |  |  |
| **conflicts_of_interest** |  |  |  |  |  |  |  |  |  |  |
| All authors / more than half | 3 | 3 | — | — |  |  |  |  |  |  |
| Less than 1/2 of the authors | 11 | 9 | 0.00 | 0.00, 0.00 | **<0.001** |  |  |  |  |  |
| None of the authors | 8 | 2 | 0.00 | 0.00, 0.00 | **<0.001** |  |  |  |  |  |
| Not reported | 10 | 5 | 0.00 | 0.00, 0.00 | **<0.001** |  |  |  |  |  |
| **PI** | 32 | 19 | 1.78 | 0.33, 9.49 | 0.49 |  |  |  |  |  |
| **IMIDS** | 32 | 19 | 3.21 | 0.58, 17.7 | 0.17 |  |  |  |  |  |
| **ANTI-CD** | 32 | 19 | 32,534,268 | 6,155,638, 171,952,703 | **<0.001** |  |  |  |  |  |
| **Transplant_eligibility** |  |  |  |  |  |  |  |  |  |  |
| Ineligible | 7 | 4 | — | — |  |  |  |  |  |  |
| All | 4 | 2 | 0.75 | 0.05, 12.1 | 0.83 |  |  |  |  |  |
| Eligible | 13 | 10 | 2.50 | 0.26, 24.3 | 0.41 |  |  |  |  |  |
| NS | 8 | 3 | 0.45 | 0.04, 4.93 | 0.50 |  |  |  |  |  |
| **Age missing** |  |  |  |  |  | 32 | 19 | 0.00 | 0.00, 0.00 | **<0.001** |
| Abbreviations: CI = Confidence Interval, OR = Odds Ratio | | | | | | | | | | |

| **TTE outcome** | | | | | | | | | | |
| --- | --- | --- | --- | --- | --- | --- | --- | --- | --- | --- |
| **Characteristic** | **Univariable models** | | | | | **Multivariable model** | | | | |
|  | **N** | **Event N** | **OR** | **95% CI** | **p-value** | **N** | **Event N** | **OR** | **95% CI** | **p-value** |
| **Publication_year** | 113 | 65 | 0.98 | 0.91, 1.05 | 0.56 |  |  |  |  |  |
| **after 2010** | 113 | 65 | 0.64 | 0.26, 1.54 | 0.31 |  |  |  |  |  |
| **after 2015** | 113 | 65 | 0.71 | 0.31, 1.61 | 0.41 |  |  |  |  |  |
| **place_of_study2** |  |  |  |  |  |  |  |  |  |  |
| Europe | 41 | 20 | — | — |  | 41 | 20 | — | — |  |
| Multinational | 49 | 36 | 2.91 | 1.18, 7.20 | **0.021** | 49 | 36 | 0.95 | 0.26, 3.48 | 0.94 |
| RoW | 7 | 5 | 2.63 | 0.41, 16.9 | 0.31 | 7 | 5 | 1.26 | 0.06, 27.9 | 0.88 |
| USA | 16 | 4 | 0.35 | 0.09, 1.36 | 0.13 | 16 | 4 | 0.24 | 0.03, 1.72 | 0.15 |
| **Pharma_reported** |  |  |  |  |  |  |  |  |  |  |
| No | 25 | 9 | — | — |  | 25 | 9 | — | — |  |
| Yes | 88 | 56 | 3.11 | 1.11, 8.73 | **0.031** | 88 | 56 | 2.43 | 0.43, 13.7 | 0.31 |
| **relapsed** |  |  |  |  |  |  |  |  |  |  |
| newly diagnosed/maintenance | 75 | 37 | — | — |  | 75 | 37 | — | — |  |
| relapsed | 38 | 28 | 2.88 | 1.15, 7.22 | **0.025** | 38 | 28 | 1.57 | 0.44, 5.61 | 0.49 |
| **Blinding_reported** |  |  |  |  |  |  |  |  |  |  |
| Open / NS | 92 | 47 | — | — |  | 92 | 47 | — | — |  |
| Double / Assessors | 21 | 18 | 5.74 | 1.54, 21.5 | **0.010** | 21 | 18 | 6.35 | 0.97, 41.8 | 0.054 |
| **Age_min** | 87 | 53 | 0.94 | 0.91, 0.98 | **0.002** |  |  |  |  |  |
| **Age_max** | 87 | 53 | 0.99 | 0.94, 1.04 | 0.67 |  |  |  |  |  |
| **Age center** | 113 | 65 | 0.90 | 0.83, 0.98 | **0.017** | 113 | 65 | 0.90 | 0.80, 1.00 | **0.045** |
| **Comparison sample size, 2-fold increase** | 113 | 65 | 1.12 | 0.74, 1.71 | 0.59 | 113 | 65 | 1.15 | 0.66, 2.02 | 0.62 |
| **Comparison_type** |  |  |  |  |  |  |  |  |  |  |
| Additional drug | 87 | 54 | — | — |  | 87 | 54 | — | — |  |
| Head-to-head | 26 | 11 | 0.45 | 0.19, 1.07 | 0.069 | 26 | 11 | 0.64 | 0.21, 1.90 | 0.41 |
| **primary_outcome3** |  |  |  |  |  |  |  |  |  |  |
| Overall survival | 20 | 3 | — | — |  | 20 | 3 | — | — |  |
| Progression | 93 | 62 | 11.3 | 3.19, 40.3 | **<0.001** | 93 | 62 | 11.5 | 3.52, 37.6 | **<0.001** |
| **follow_up** | 111 | 64 | 1.00 | 0.99, 1.00 | 0.32 |  |  |  |  |  |
| **conflicts_of_interest** |  |  |  |  |  |  |  |  |  |  |
| All authors / more than half | 43 | 28 | — | — |  |  |  |  |  |  |
| Less than 1/2 of the authors | 36 | 24 | 1.07 | 0.33, 3.49 | 0.91 |  |  |  |  |  |
| None of the authors | 18 | 6 | 0.27 | 0.06, 1.12 | 0.070 |  |  |  |  |  |
| Not reported | 16 | 7 | 0.42 | 0.12, 1.46 | 0.17 |  |  |  |  |  |
| **PI** | 113 | 65 | 1.71 | 0.71, 4.12 | 0.23 |  |  |  |  |  |
| **IMIDS** | 113 | 65 | 0.95 | 0.42, 2.15 | 0.91 |  |  |  |  |  |
| **ANTI-CD** | 113 | 65 | 39,272,134 | 20,100,949, 76,727,747 | **<0.001** |  |  |  |  |  |
| **Transplant_eligibility** |  |  |  |  |  |  |  |  |  |  |
| Ineligible | 32 | 13 | — | — |  |  |  |  |  |  |
| All | 9 | 4 | 1.17 | 0.42, 3.22 | 0.76 |  |  |  |  |  |
| Eligible | 25 | 17 | 3.11 | 0.87, 11.1 | 0.081 |  |  |  |  |  |
| NS | 47 | 31 | 2.83 | 1.04, 7.73 | **0.042** |  |  |  |  |  |
| **Age missing** |  |  |  |  |  | 113 | 65 | 0.08 | 0.00, 6.98 | 0.26 |
| Abbreviations: CI = Confidence Interval, OR = Odds Ratio | | | | | | | | | | |
